# The prognostic value of comorbidity for the severity of COVID-19: A systematic review and meta-analysis study

**DOI:** 10.1371/journal.pone.0246190

**Published:** 2021-02-16

**Authors:** Mobina Fathi, Kimia Vakili, Fatemeh Sayehmiri, Ashraf Mohamadkhani, Mohammadreza Hajiesmaeili, Mostafa Rezaei-Tavirani, Owrang Eilami

**Affiliations:** 1 Student Research Committee, Faculty of Medicine, Shahid Beheshti University of Medical Sciences, Tehran, IR Iran; 2 Faculty of Medicine, Shahid Beheshti University of Medical Sciences, Tehran, IR Iran; 3 Digestive Disease Research Center, Tehran University of Medical Sciences, Tehran, IR Iran; 4 Anesthesia and Critical Care Department, Critical Care Quality Improvement Research Center, Loghman Hakim Hospital, Shahid Beheshti University of Medical Sciences, Tehran, Iran; 5 Faculty of Paramedical Sciences, Proteomics Research Center, Shahid Beheshti University of Medical Sciences, Tehran, IR Iran; 6 Department of Family Medicine, Shiraz University of Medical Science, Fars, IR Iran; Jouf University, Kingdom of Saudi Arabia, SAUDI ARABIA

## Abstract

**Background and objectives:**

With the increase in the number of COVID-19 infections, the global health apparatus is facing insufficient resources. The main objective of the current study is to provide additional data regarding the clinical characteristics of the patients diagnosed with COVID-19, and in particular to analyze the factors associated with disease severity, lack of improvement, and mortality.

**Methods:**

102 studies were included in the present meta-analysis, all of which were published before September 24, 2020. The studies were found by searching a number of databases, including Scopus, MEDLINE, Web of Science, and Embase. We performed a thorough search from early February until September 24. The selected papers were evaluated and analyzed using Stata software application version 14.

**Results:**

Ultimately, 102 papers were selected for this meta- analysis, covering 121,437 infected patients. The mean age of the patients was 58.42 years. The results indicate a prevalence of 79.26% for fever (95% CI: 74.98–83.26; I^2^ = 97.35%), 60.70% for cough (95% CI: 56.91–64.43; I^2^ = 94.98%), 33.21% for fatigue or myalgia (95% CI: 28.86–37.70; I^2^ = 96.12%), 31.30% for dyspnea (95% CI: 26.14–36.69; I^2^ = 97.67%), and 10.65% for diarrhea (95% CI: 8.26–13.27; I^2^ = 94.20%). The prevalence for the most common comorbidities was 28.30% for hypertension (95% CI: 23.66–33.18; I^2^ = 99.58%), 14.29% for diabetes (95% CI: 11.88–16.87; I^2^ = 99.10%), 12.30% for cardiovascular diseases (95% CI: 9.59–15.27; I^2^ = 99.33%), and 5.19% for chronic kidney disease (95% CI: 3.95–6.58; I^2^ = 96.42%).

**Conclusions:**

We evaluated the prevalence of some of the most important comorbidities in COVID-19 patients, indicating that some underlying disorders, including hypertension, diabetes, cardiovascular diseases, and chronic kidney disease, can be considered as risk factors for patients with COVID-19 infection. Furthermore, the results show that an elderly male with underlying diseases is more likely to have severe COVID-19.

## Introduction

On December 31, 2019, a cluster of cases of pneumonia with an unknown source was reported in Wuhan, China, related to the Huanan seafood wholesale market [1, 2]. Subsequently, on January 9, 2020, the Chinese Center for Disease Control and Prevention announced its causative factor as the novel severe acute respiratory syndrome coronavirus 2 (SARS-CoV-2). This global disorder, caused by SARS-CoV-2, became known as COVID-19. Just two months later, on March 11, 2020, the Director General of the World Health Organization (WHO) designated the spread of COVID-19 as a pandemic [3, 4].

As of October 15, 2020, a total of 38,394,169 confirmed COVID-19 patients and 1,089,047 dead cases have been reported by WHO globally, and as of the time of this writing, the number of patients is increasing. In addition, 235 countries, regions, or territories with cases of COVID-19 infection have been identified worldwide [5]. The number of COVID-19 infected patients is increasing worldwide, and due to its correlation with a high morbidity and mortality rate, this trend has caused a new global phobia called Coro-phobia [6]. According to the results of a number of studies, COVID-19 infection can be diagnosed based on several symptoms, including cough, fever, fatigue, diarrhea, myalgia, and dyspnea [7–9].

Huang et al. evaluated the clinical features of 41 cases with COVID-19 infection, and their results showed that 13 (32%) cases had comorbidities, such as hypertension, chronic obstructive pulmonary disease, diabetes, and cardiovascular disease [10]. In addition, Guan et al. reported the results of 1,099 cases of COVID-19 infection, indicating that 261 (23.7%) cases had underlying diseases [11]. These studies and similar investigations show that some complications can be considered as risk factors for negative outcomes in patients with COVID-19 infection [12].

Furthermore, Lai et al. [13] realized that the mortality rate was associated with the country’s healthcare resources. However, in many countries, the intensive care units (ICUs) and the invasive ventilators are not enough to treat critically-infected patients. Consequently, clinical operators should consider the risk factors for critical cases of COVID-19, correctly assign medical resources, identify severe patients in the early stages of the disease, and devise an appropriate treatment plan to reduce the mortality rate and improve the effectiveness of the treatment [11]. Therefore, knowing the risk factors and the underlying diseases in COVID-19 patients is important for healthcare professionals, especially for immunocompromised people and the elderly. Subsequently, the evaluation of the prevalence of chronic diseases, such as hypertension, kidney disease, cardiovascular disease, and diabetes, is considered as one of the most important measures to reduce negative outcomes in COVID-19 patients [14].

Currently, we have no approved vaccines or curative drugs against the new COVID-19 infection; hence, to prevent COVID-19 infection, a systematic review and meta-analysis has been conducted to study the prevalence of common comorbidities by analyzing studies focusing on clinical risk factors for COVID-19.

## Materials and methods

### Study selection

We searched PubMed, Scopus, Web of Science, and Embase databases to identify relevant studies that were published on COVID-19 until September 24, 2020. The keywords for our search included: Kidney, COVID, Hypertension, Diabetes, ((Heart) OR Cardio-), and Comorbidities. Moreover, the citations in the identified studies were also scanned in order to identify references related to the subject matter.

### Inclusion and exclusion criteria

All studies evaluating the clinical risk factors, underlying diseases, and comorbidities of COVID-19 have been reviewed. For our final analysis, the studies selected should report data on the prevalence of each of the risk factors for the severity of COVID-19 and its comorbidities in patients with COVID-19 infection, including hypertension, chronic kidney disease, diabetes, cardiovascular and cerebrovascular diseases, and coronary heart disease. Only human investigations were selected. The full texts of the relevant studies were assessed based on the inclusion and exclusion criteria. Studies that did not have adequate data, non-human studies, duplicate publications, and non-English publications (except for those with useful abstract data) were excluded.

### Data extraction

All the selected papers were independently evaluated and screened by two authors (M.F. and K.V.), and any disagreements were resolved through discussion. The following data were collected for the final analysis: prevalence of hypertension, chronic kidney disease, diabetes, cardiovascular and cerebrovascular diseases, and coronary heart disease, along with the prevalence of symptoms such as fever, dyspnea, diarrhea, cough, fatigue, and myalgia.

### Statistical analysis

Because the effect size in this study was based on proportion (i.e., prevalence of hypertension, chronic kidney disease, diabetes, cardiovascular and cerebrovascular diseases, and coronary heart disease), binomial distribution was employed to calculate the variance for each study. The prevalence in various studies were combined using the average weight. An inverse relationship between the variance of the study and its weight was observed. The *I*^*2*^ index was applied to evaluate the heterogeneity. In the case of heterogeneous studies, the random effects model were used. To analyze the data, Stata software application (version 14) was used. The Metaprop (meta-analysis for proportion) was used in Stata when p was close to 0 or 1. Moreover, to stabilize the variances, we used Freeman-Tukey Double Arcsine Transformation [15]. This study was performed with the approval of the Ethics Committee of Shahid Beheshti University of Medical Sciences (*IR.SBMU.RETECH.REC*.1399.084).

### Publication bias

Publication bias was graphically defined by the Funnel plot. This funnel plot is a scatter plot with 2 variates (*x*, y) that evaluates the research estimate of the effect size against its sample size. A linear regression analysis was conducted for publication bias, which includes both slope and intercept parameters. It was calculated based on the following equation:

yi=α+βxi+εi (Eq 1)

i = 1… r (r = the number of studies), yi = standardized estimate, xi = precision of studies, εi = error terms

### Quality assessment

The Newcastle-Ottawa Scale (NOS) was employed to assess the quality of each included paper [16]. This scale includes 8 assessment items for evaluating the quality of studies, including ‘selection’, ‘comparability’, and ‘outcome’, based on the Ottawa checklist for the cross-sectional studies. Based on the score standard in NOS, cross-sectional studies can be classified as Very Good Studies (9–10 points), Good Studies (7–8 points), Satisfactory Studies (5–6 points), and Unsatisfactory Studies (0 to 4 points) (**Table 1 in S1 File**).

## Results and discussion

### Study selection

**Fig 1:**
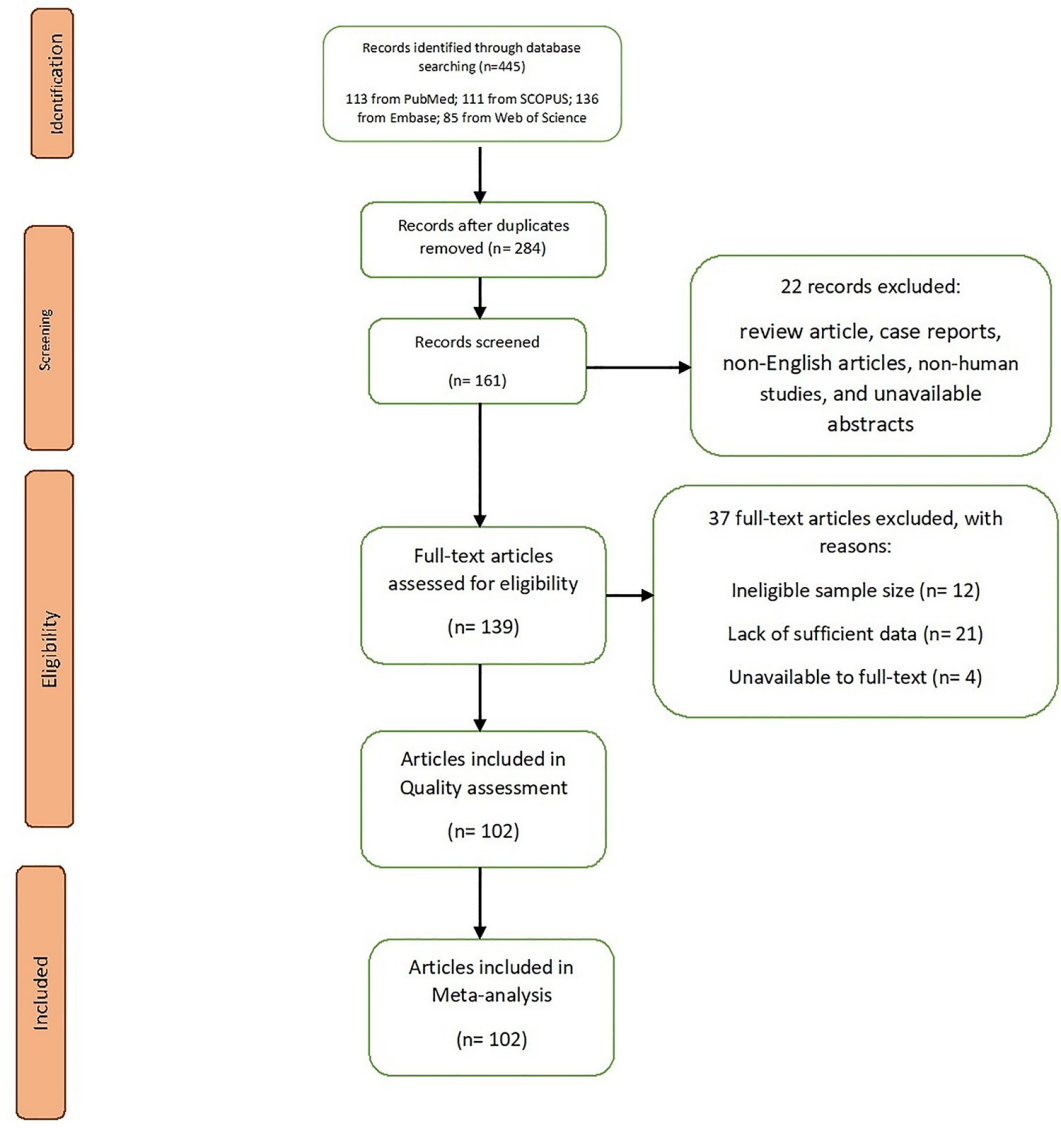
Study flow diagram.

The current study was carried out based on the PRISMA checklist [17]. It should be noted that initially, 113 studies were identified through primary search on PubMed, 111 were identified by searching Scopus, 136 were identified by searching Embase, and 85 were identified by searching Web of Science. However, 284 of those 445 articles were excluded because of duplication. After screening the titles and abstracts of all identified papers, 22 more studies were excluded due to being review articles, case reports, and non-English articles, or due to unavailable abstracts. The full texts of the 139 remaining studies were reviewed, and 37 studies were excluded due to ineligible sample size (n = 12), lack of sufficient data (n = 21), or unavailable full text (n = 4). Finally, 102 articles, published from February, 2020 to July, 2020, were selected for the meta-analysis (**Fig 1, Table 1**).

**Table 1:** Baseline characteristics of studies included in this meta-analysis.

study	Place	Median age	Exposure history (No.)	Patients (No.)			Symptoms (No.)					Comorbidities (No.)					Mortality rate (%.)	Total Score	Total Quality
				All	Male	Female	Fever	Cough	Fatigue or Myalgia	Diarrhea	Dyspnea	All	Hypertension	Diabetes	Cardiovascular disease	Chronic kidney disease			
[18]	China	56	73	191	119	72	180	151	44	9	56	91	58	36		2	28.27	10	Very Good
[19]	China		3	85	62	23	78	19	50	16	50	58	32	19		3		8	Good
[20]	China	57.00		140	71	69	110	90	90	18	44	90	42	17		2		9	Very Good
[21]	China	59.00		174	76	98	136	56	47	21	42		43	37	32	13		9	Very Good
[22]	China			1590	904	674	1351	1052	584	57	331	399	269	130	59	269	3.10	8	Good
[23]	China	51.00	99	201	128	73	188	163	65		80	66	39	22	8	2	21.90	9	Very Good
[24]	China	69.00		339	166	173	311	179	135	43	138		138	54	53	13	19.17	9	Very Good
[25]	China	73.00		25	10	15					23	25	16	10	8	5	100.00	5	satisfactory
[26]	U.S			7162								2692		784	647	213		6	satisfactory
[27]	U.S			180			153	155	62	48	144	159	79	47	45	20		9	Very Good
[28]	China	50.00	311	1012	524	488	761	531	170	152	231	114	46	27	15			9	Very Good
[1]	China	49.00	27	41	30	11	40	31	18	1	22	13	6	8	6		14.63	8	Good
[29]	China			99	67	32	82	81	1	2	31	50					11.00	7	Good
[12]	China	56.00	12	138	75	63	136	82	96	14	43	64	43	14	20	4	4.30	9	Very Good
[30]	China			17	9	8	12	13	4	2		3	1					7	Good
[11]	China	47.00		1099	637	459	473	745	419	42	205	261	165	81		8	1.40	8	Good
[31]	China	41.00	23	62	35	27	48	50	32	3		20	5	1		1	0.00	6	satisfactory
[32]	China		0	80	39	41	63	30	18	1	30	38				1	0.00	7	Good
[33]	China	57.00	0	137	61	76	112	66	44	11	26	27	13	14	10		11.68	8	Good
[34]	China	40.00		61	31	30	60	39	35	6	3		12	5	1		0.00	9	Very Good
[35]	China			52	35	17	51	40	6		33	21		9	5		61.54	6	satisfactory
[36]	China			187	91	96							61	28		6	23.00	9	Very Good
[37]	Italy			355										72			100.00	7	Good
[38]	China			12	8	4	10	11	4	2			3	2	4	2		6	satisfactory
[39]	China			9	5	4	8	5	4			1	0	1	0	0		3	Unsatisfactory
[40]	China		31974	44672	22981	21691							2683	1102	873		2.30	8	Good
[10]	China	38.00	0	78	39	39		34					8	5				7	Good
[41]	China	63.00		701	367	334	213					297	233	100		14	16.10	9	Very Good
[42]	China	57.00		193	95	98	171	134	75	35	70	105					66.32	10	Very Good
[43]	China	62.00		274	171	103	249	185	137	77	120	133	93	47	23	4	41.24	9	Very Good
[44]	China	72.50		82	54	28	64	53	38	10	52	62	46	15	17			8	Good
[45]	China			5	4	1	5	5	3		5		2	1				8	Good
[46]	China	64.00		416	205	211	334	144	55	16	117		127	60				10	Very Good
[47]	China	54.00		116	67	49							43	18		5		9	Very Good
[48]	US			21			11	11			17	18		7		10	52.40	7	Good
[49]	China	54.00		203	108	95	181	122	16	10	3	88	43	16	16	8	12.80	9	Very Good
[50]	China	50.00		91	37	54	65	55	40	21	3		15	8			0.00	6	satisfactory
[51]	China	58.00		452	235	217	423	152	212	122	232	201	135	75	27	10	36.70	9	Very Good
[52]	China			69	32	37	30	38	29	10	20		9	7	8		7.50	8	Good
[53]	China	55.00	8	221	108	113	200	136	156	25	64	78	54	22	22		5.40	9	Very Good
[54]	China	50.00		90	39	51	70	57	19	5		45	17	5	3			8	Good
[55]	China			109	74	35	99	77	58	29	75		65	34				9	Very Good
[56]	China	54.00	47	102	53	49	83	50	56	11	35	47	28	11	5	4	16.70	9	Very Good
[57]	Italy	63.00		1591	1304	287						1043	509	180	223	36		9	Very Good
[58]	US	63.00		5700	3437	2263						5700	3026	1808		268	21.00	9	Very Good
[59]	China	68.00		54									24	13			48.10	6	satisfactory
[60]	China			200	99	101						161	101	137	16		17.00	10	Very Good
[61]	China	64.91		47	26	21					12		18		5			6	satisfactory
[62]	China			110	60	50							57	19	16	9		9	Very Good
[63]	China	61.00		323	166	157	271	164			14		105	47		7		9	Very Good
[64]	China			49	31	18	39	17	9	4	6	16						6	satisfactory
[65]	China	47.00		298	149	149	192	54	6	6			38	19	11			8	Good
[66]	China			265			220	131	67	17	5		52	21	14			9	Very Good
[67]	China	45.00		242	119	123	162	132	72	15	21		36	15	9		0.80	10	Very Good
[68]	China	48.00		84	48	36	54	43	16	6	3		12	10	5			8	Good
[69]	China	55.00		109	59	50	90	67	62	12			37	12	7	10	28.40	9	Very Good
[70]	China	60.00		548	279	269	476	415	258	179	310		166	83		10	16.50	9	Very Good
[71]	China	52.00		113	66	47	91						26	9			1.80	9	Very Good
[72]	China	60.00		27	12	15	21	16	3		11	13	5	6	3		37.00	8	Good
[73]	China			136	58	78	115	85	24		17	75	53	24	6	2	0.00	9	Very Good
[73]	China			652	349	303	521	421	115		20	143	73	33	5	5	0.00	9	Very Good
[74]	Bolivia	39.00		12	6	6	9	9	5	2		2	1				0.00	6	Satisfactory
[75]	China	45.00	104	161	80	81	122	101	64	17	23	33	22	7			0.00	9	Very Good
[76]	China	47.00	113	135	72	63	120	102	44	18	18	43	13	12	7		0.70	9	Very Good
[77]	China			37	14	23	15	12			0	10	8	4			0.00	6	Satisfactory
[78]	China			214	87	127	132	107		41		83	51	30	15	6	0.00	9	Very Good
[79]	China	65.00		112	57	55	98	79			63		36	19			0.00	9	Very Good
[80]	China			136	90	46	52	71	66	27	102		41	27		3	0.00	8	Good
[81]	China	70.00		25	19	6						16	12	6	8		100.00	8	Good
[81]	China	51.00		149	60	89						36	25	11	8		0.00	8	Good
[82]	Singapore	47.00		18	9	9	13	15		3	2	5	4	1				5	unsatisfactory
[83]	Korea	75.50		54	33	21						49		16	32	5	100.00	7	Good
[84]	China			92	49	43						65	51	13	16	2	100.00	7	Good
[85]	China			168	86	82	156	121	65	44	59	57	41	20	9	2	10.10	10	Very Good
[86]	China			13	10	3	13	10	8	2	12		2	1			38.50	7	Good
[86]	China			12	2	10	11	7	8	3	8		0	0			0.00	7	Good
[87]	UK	82.00		101	64	37	9	4	9	3	67		54	36	18	21	74.20	8	Good
[88]	China		2	3	2	1	1	2	1	2			0	1		0	0.00	6	satisfactory
[89]	China		134	149	81	68	114	87		11	2	52			28		0.00	7	Good
[90]	China		31	81	42	39	59	48	7	3	34	21	12	10	8	3	4.00	8	Good
[91]	Korea	77.00		66	37	29						61	30	23	10	5		9	Very Good
[92]	China	52.00		108	43	65	80	84	28	8	15	25	16	5	4		11.10	9	Very Good
[93]	China	53.00	425	476	271	205	390	269			109	205	113	49	38	4	8.00	9	Very Good
[94]	China	54.00	37	155	86	79	126	97	60	7	50		37	15	15	6	0.00	9	Very Good
[95]	China		186	487	259	228							99	29	11	7	0.00	9	Very Good
[96]	USA	72		167	55	112							74	38	68	43	21	8	Good
[97]	Italy	65		410	229	111							203	69		47		10	Very Good
[98]	USA	45		100	56	44	71	87	21	10	38		19	10		6		8	Good
[99]	Italy			99	80	19	39						63	30		15		9	Very Good
[100]	USA	69		105	53	52	49	76	51	24	56		62	35	40	27	33	9	Very Good
[101]	Greece			21	17	4							7	2	0	0		9	Very Good
[101]	Greece			24	18	6							11	4	3	1		9	Very Good
[101]	Greece			45	37	8							27	11	16	3		9	Very Good
[102]	Italy	68		236	177	59	230	144			83			37	127	15		10	Very Good
[103]	USA	52.7		13442	7841	5957							3043	2710	2086	809		9	Very Good
[104]	China			83	44	39	72	65	15	7	9	15	5	7	1			8	Good
[105]	China			296	140	156	213	197					42	30			6.4	9	Very Good
[106]	China			1590	904	674	1351	1052	584	57	331	399	269	130	59			8	Good
[107]	Italy	68.00		6272	3968	2303							3631	859	1891	181		9	Very Good
[108]	USA			18472	7388	11083							7388	3509	2216			10	Very Good
[109]	UK			1200	686	513							645	417	160	206		10	Very Good
[110]	USA	73.00		206	101	105							152		206	7131		10	Very Good
[5]	Spain	69.10		1139	695	444							617	310	312	89		9	Very Good
[111]	Belgium	86.00		154	51	103							39	28		181		6	Satisfactory
[112]	USA	52.70		442	256	186							159	84	47			9	Very Good
[113]	UK	68.00		605	272	333							200	30	90	206		10	Very Good
[114]	UK	69.30		1474	787	686							728	228	138	7131		8	Good

*Abbreviations: No = Number.

### Demographic characteristics

Based on the data analysis, the total number of hospitalized patients covered by this analysis was 121,437, which includes 27,062 patients with hypertension, 14,300 patients with diabetes, 9,914 patients with cardiovascular diseases, 9,603 patients with chronic kidney disease, 266 patients with coronary heart disease, and 176 patients with both cardiovascular and cerebrovascular diseases. According to the results, 54.92 percent of the subjects were male (95% CI: 52.92–56.92; I^2^ = 96.53%), while 45.12 percent were female (95% CI: 43.12–47.12; I^2^ = 96.55%) (**Table 2**). The mean age of the subjects in all the included studies was 58.42 years. Most of the studies were conducted in China, while 11 were conducted in the United States, 6 in Italy, 4 in the UK, 2 in Korea, 1 in Spain, 1 in Belgium, 1 in Greece, 1 in Bolivia, and 1 in Singapore. We should also note that six selected studies have not reported the number of patients with both cardiovascular and cerebrovascular disease separately. Based on the papers that reported the number of individuals with a history of exposure, the prevalence of the total exposure history was calculated as 35.56% (95% CI: 21.97–50.38; I^2^ = 99.43%) (**Table 2**).

**Table 2:** Statistical analysis of reviewed articles.

	Number of studies	Prevalence (%)	95% CI	I^2^(%)
**Sex**				
male	101	54.92	(52.92–56.92)	96.53
female	101	45.12	(43.12–47.12)	96.55
**Exposure history**	21	35.56	(21.97–50.38)	99.43
**Signs and symptoms**				
Fever	73	79.26	(74.98–83.26)	97.35
Cough	71	60.70	(56.91–64.43)	94.98
Fatigue or Myalgia	60	33.21	(28.86–37.70)	96.12
Dyspnea	61	31.30	(26.14–36.69)	97.67
Diarrhea	56	10.65	(8.26–13.27)	94.20
**Comorbidity**	54	45.98	(34.47–57.71)	99.69
Hypertension	96	28.30	(23.66–33.18)	99.58
Cardiovascular and cerebrovascular diseases	6	18.25	(7.47–32.11)	94.10
Diabetes	98	14.29	(11.88–16.87)	99.10
Chronic kidney disease	58	5.19	(3.95–6.58)	96.42
***Heart diseases***				
Cardiovascular disease	68	12.30	(9.59–15.27)	99.33
Coronary heart disease	17	7.37	(4.86–10.28)	86.50
**Mortality**	58	8.91	(6.34–11.84)	98.45

### Clinical manifestations

The results of this analysis indicate that the prevalence of the most common symptoms of COVID-19 were 79.26% for fever (95% CI: 74.98–83.26; I^2^ = 97.35%), 60.70% for coughing (95% CI: 56.91–64.43; I^2^ = 94.98%), 33.21% for fatigue or myalgia (95% CI: 28.86–37.70; I^2^ = 96.12%), 31.30% for dyspnea (95% CI: 26.14–36.69; I^2^ = 97.67%), and 10.65% for diarrhea (95% CI: 8.26–13.27; I^2^ = 94.20%) (**Table 2**).

### Comorbidities

**Fig 2:**
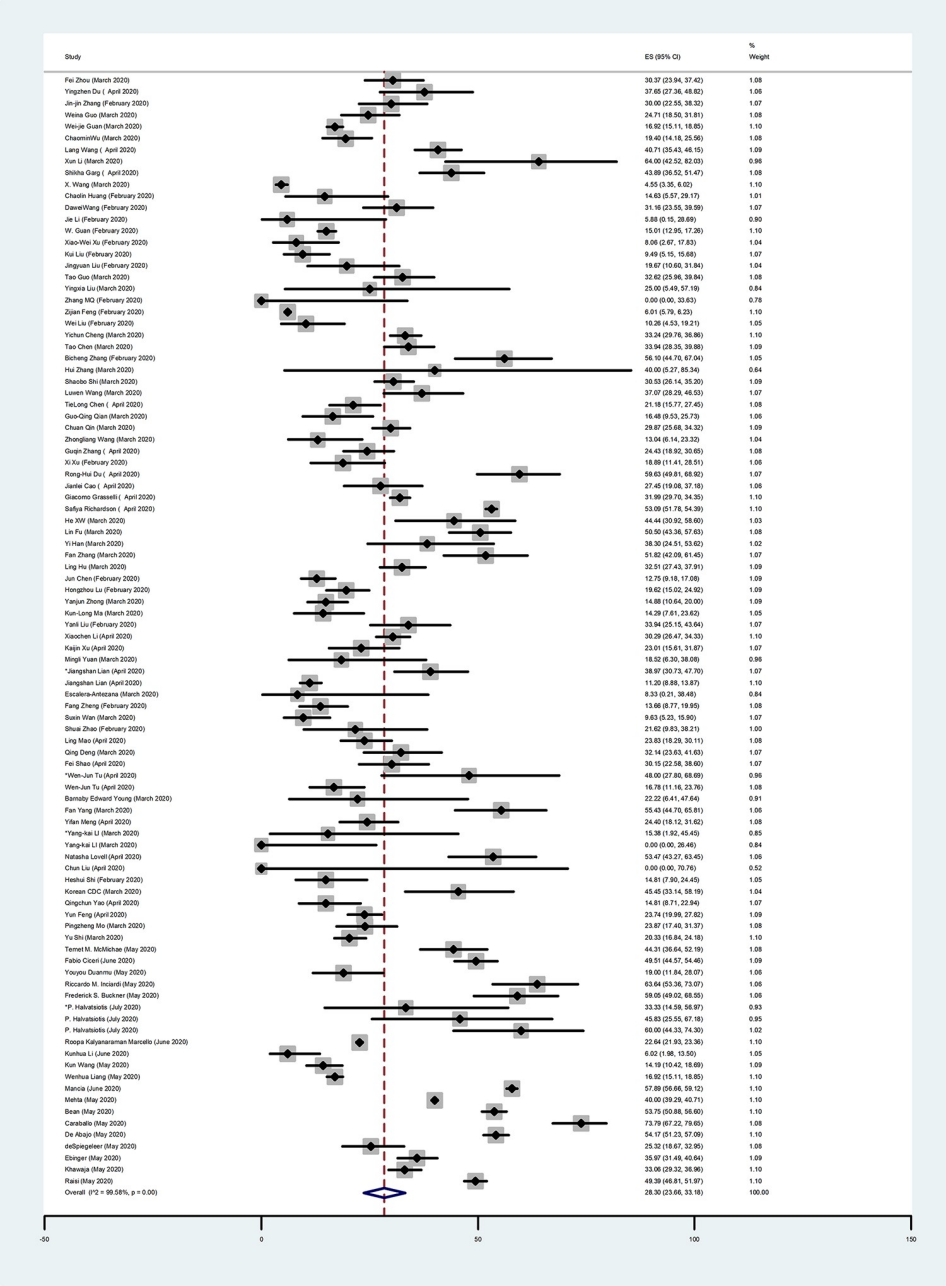
Forest Plot of the prevalence of hypertension in COVID-19 patients. Each square indicates the effect estimate of individual articles with their 95% CI Size of squares is proportional to the weight of each paper in the meta-analysis. In this plot, papers are indicated in the order of first author’s names and publication date (based on a random effects model).

**Fig 3:**
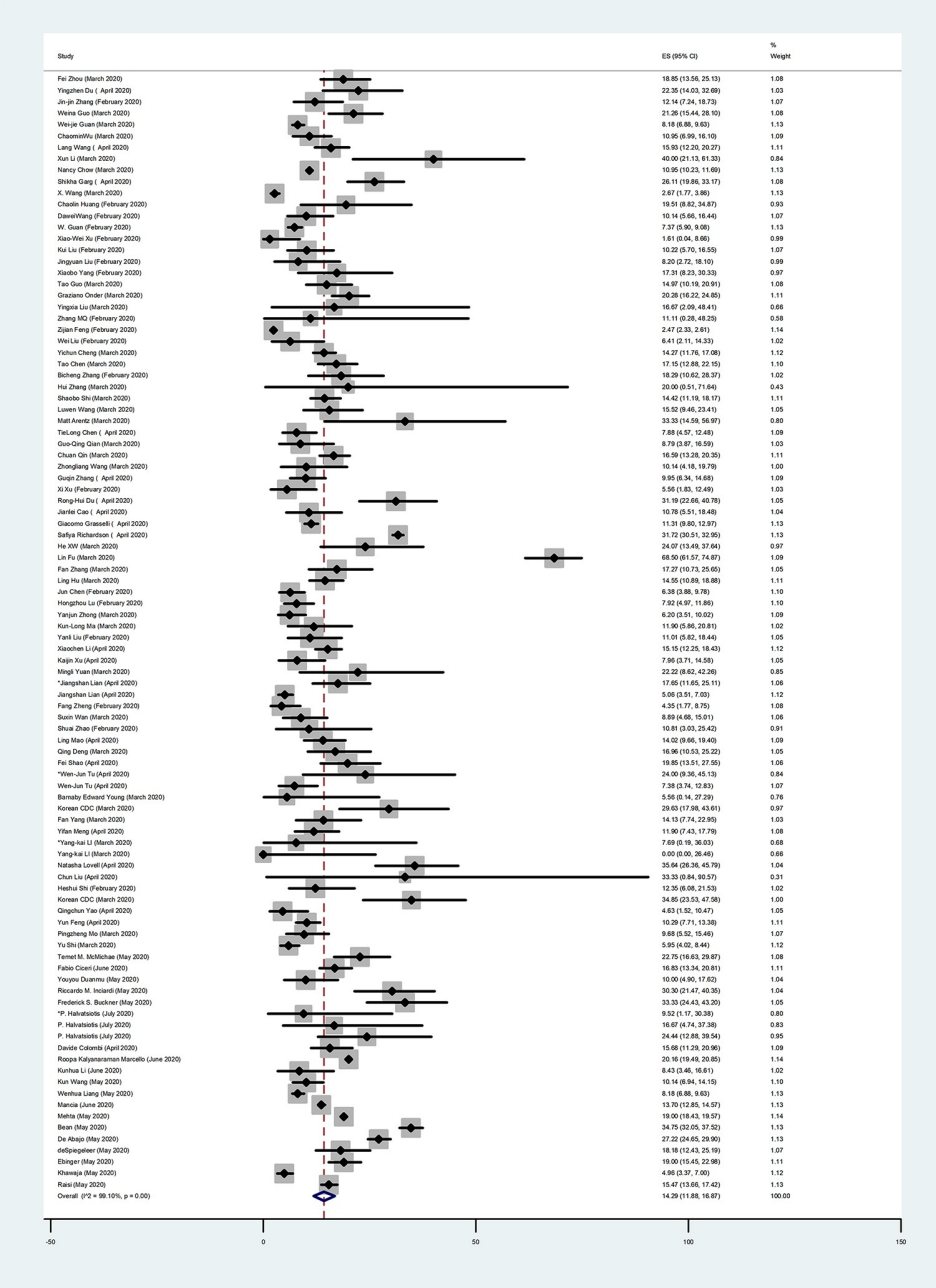
Forest Plot of the prevalence of diabetes in COVID-19 patients. Each square indicates the effect estimate of individual articles with their 95% CI Size of squares is proportional to the weight of each paper in the meta-analysis. In this plot, papers are indicated in the order of first author’s names and publication date (based on a random effects model).

**Fig 4:**
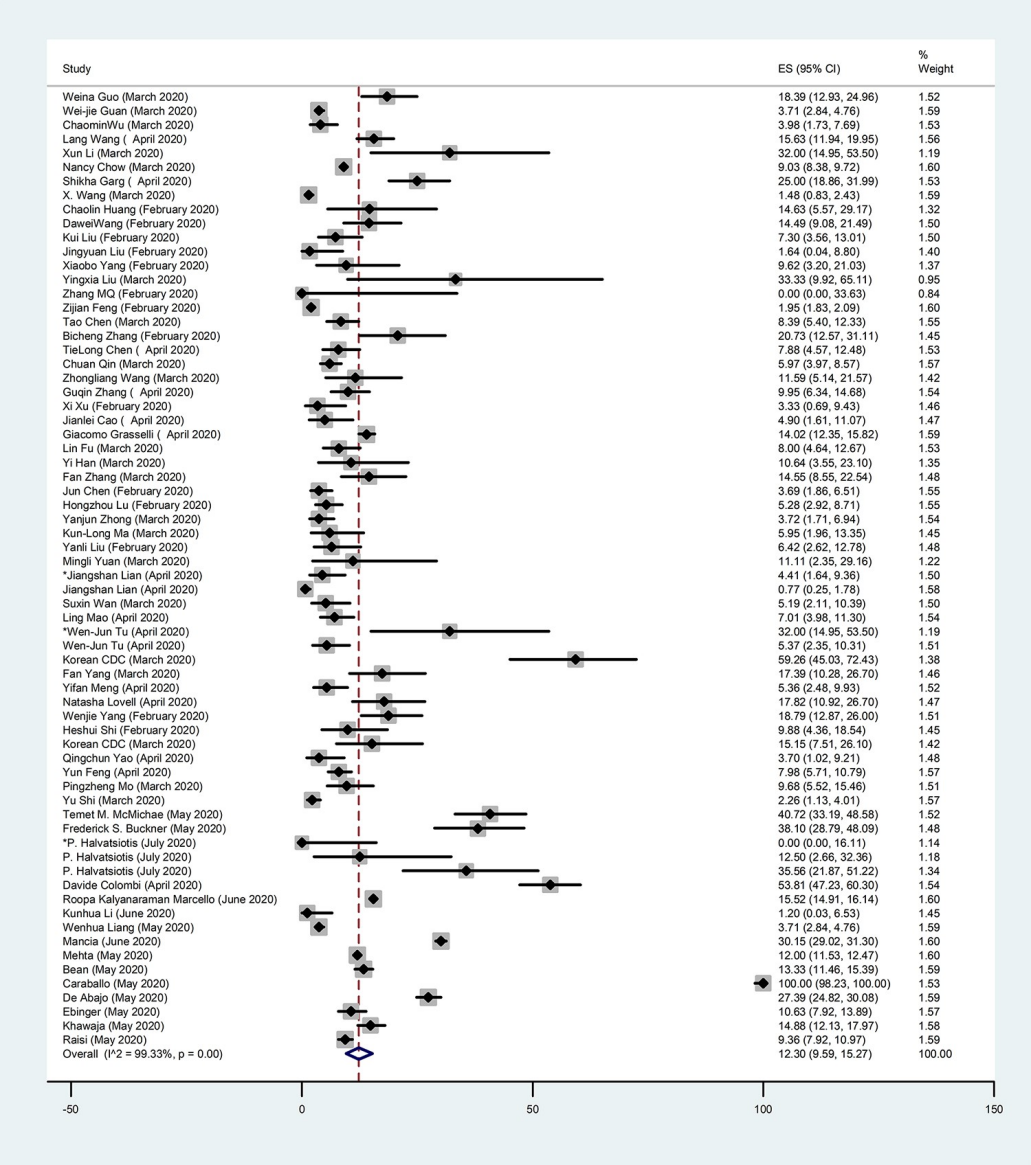
Forest Plot of the prevalence of cardiovascular diseases in COVID-19 patients. Each square indicates the effect estimate of individual articles with their 95% CI Size of squares is proportional to the weight of each paper in the meta-analysis. In this plot, papers are indicated in the order of first author’s names and publication date (based on a random effects model).

In this systematic review, we focused on the prevalence of people with comorbidities, which was 45.98% (95% CI: 34.47–57.71; I^2^ = 99.69%) (**Table 2**). The prevalence of the most common comorbidities were 28.30% for hypertension (95% CI: 23.66–33.18; I^2^ = 99.58%) (**Fig 2, Table 2**), 14.29% for diabetes (95% CI: 11.88–16.87; I^2^ = 99.10%) (**Fig 3, Table 2**), 12.30% for cardiovascular diseases (95% CI: 9.59–15.27; I^2^ = 99.33%) (**Fig 4, Table 2**), and 5.19% for chronic kidney disease (95% CI: 3.95–6.58; I^2^ = 96.42%) (**Table 2)**. We also assessed the prevalence of some other comorbidities found in our included articles, such as cardiovascular and cerebrovascular diseases with 18.25% (95% CI: 7.47–32.11; I^2^ = 94.10%), and coronary heart disease with 7.37% (95% CI: 4.86–10.28; I^2^ = 86.50%) (**Table 2**).

### Outcomes

We calculated the percentage of the mortality rate based on all the selected studies reporting mortality cases, and it was 15.18% (95% CI: 10.88–20.02; I^2^ = 99.19%). After performing the sensitivity analysis and eliminating five studies from our selected studies because they were conducted on deceased individuals, i.e., the outlier studies, the overall mortality rate was calculated based on the remaining 58 studies as 8.91% (95% CI: 6.34–11.84; I^2^ = 98.45%) (**Table 2**).

### Publication bias

**Fig 5:**
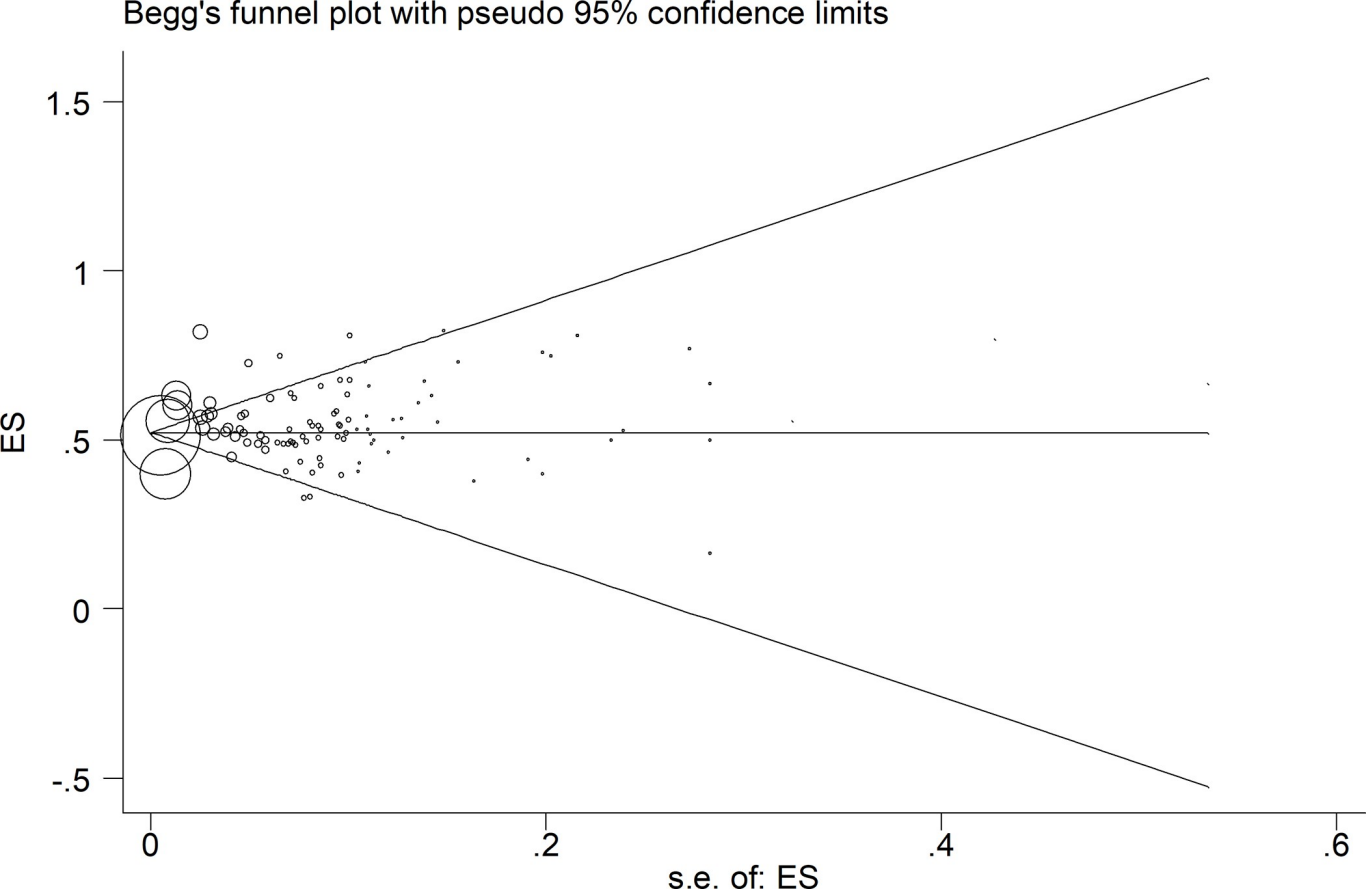
Begg’s funnel plot for publication bias.

**Fig 6:**
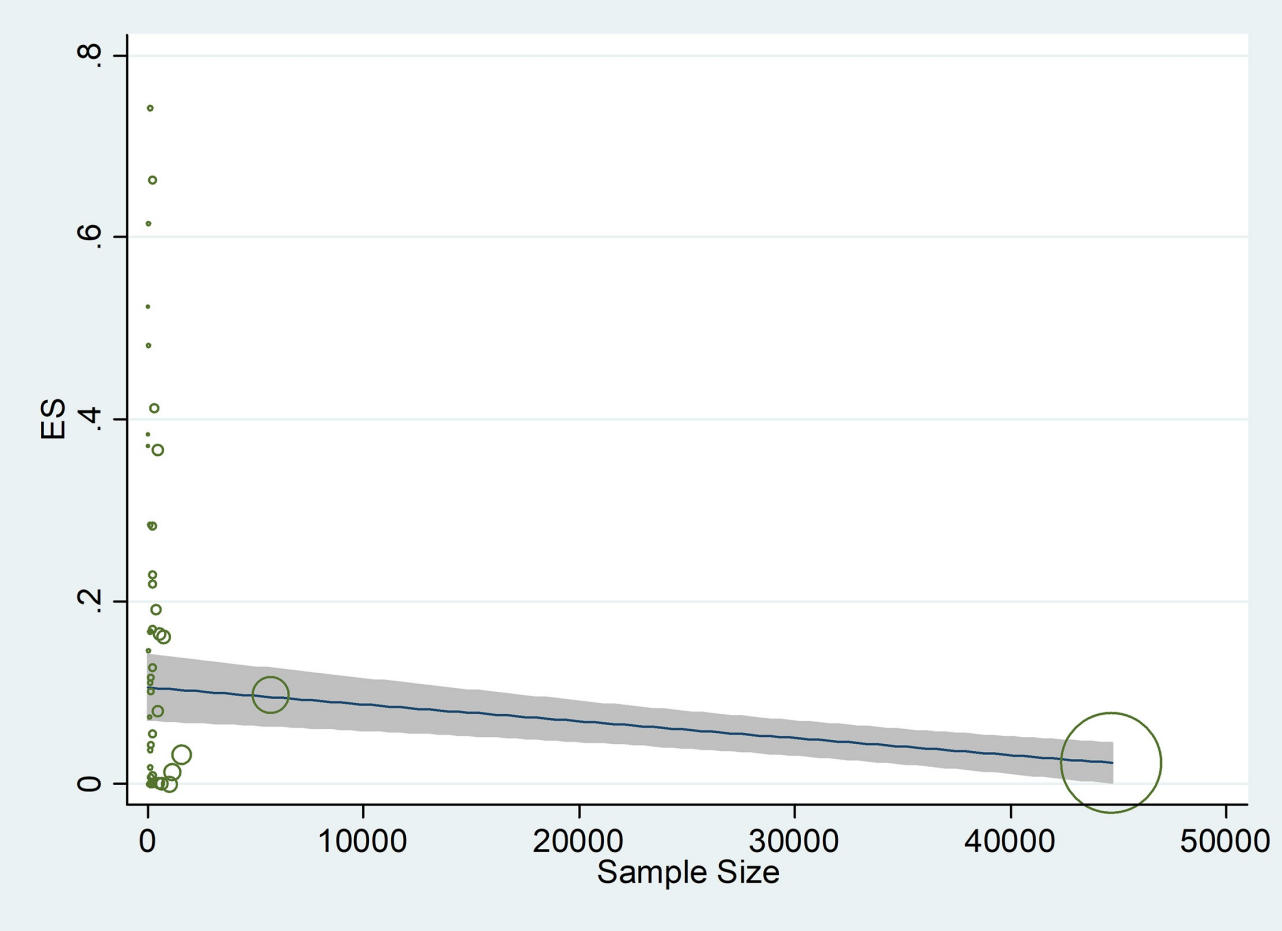
The association between prevalence of risk factors for COVID-19 and sample size, using Meta regression.

**Fig 5** Shows the Begg’s funnel plot of the comorbidity studies performed on patients with COVID-19 infection. The interpretation of our Begg’s funnel plot (p = 0.780), as well as Egger’s test (p = 0.069), shows no sign of publication bias in the included studies. Therefore, it is understandable that reports have been published with both positive and negative outcomes (**Fig 5**). Based on the results of the meta-regression analysis, the correlation between the prevalence and the sample size was evaluated. Based on this assessment and the figure, there was no significant relation between the prevalence and the sample size (P = 0.428). It should be noted that in this figure, the circles indicate the weight of the papers (**Fig 6**).

In December 2019, a new coronavirus disease, i.e., coronavirus disease 2019 (COVID-19), spread globally [115]. The city of Wuhan in China is considered to be one of the most infected areas in the world [116]. Because of this epidemic contagious disease, aggressive quarantine has been enforced by the government, which has played an important role in monitoring the prevalence of this virus. COVID-19 is not only highly contagious and insidious, but it also has the ability to give rise to cluster outbreaks [117, 118]. The SARS-CoV-2 virus can attack certain cells in the body, particularly alveolar epithelial cells, through a specific enzyme called the angiotensin-converting enzyme 2 (ACE2). ACE2 is considered to be the isoenzyme of ACE, which is mainly distributed in the testes, the kidneys, the colon, the cardiovascular system, the lungs, and other organs [119].

As of the time of writing this review, the pathogenesis and origin of COVID-19 have remained unknown, and no useful medical treatment has been offered for COVID-19 infection. Recommendations insist on intensive supportive care to reduce the risk of infection with this SARS-CoV-2 virus [10, 29]. Unfortunately, in severely-ill COVID-19 patients, the disorder develops rapidly, and the acute respiratory distress syndrome (ARDS), as one of the worst complications of COVID-19 outcomes, can occur earlier and even lead to death. In a study by Tian et al. [120], it was stated that the percentage of mild and seriously-ill patients was 73% and 18%, respectively. Furthermore, Wang et al. found that the risk of exacerbation in patients with cerebrovascular disease, hypertension, cardiovascular disease, or diabetes was higher than others [1].

This meta-analysis is based on data from 102 studies with laboratory-confirmed SARS-CoV-2 cases. The selected studies were from all over the world, and not a specific region. Based on previous studies on SARS-CoV and MERS-CoV, it is understood that these viruses affect males more than females [121, 122]. Moreover, based on our results, men with 54.92% (95% CI: 52.92–56.92; I^2^ = 96.53%) are more affected by SARS-CoV-2 than women with 45.12% (95% CI: 43.12–47.12; I^2^ = 96.55%). Furthermore, this may be due to women’s more adaptive innate immune system and robust responses [48]. Another reason for our findings may be that men have a tendency to have more harmful lifestyle habits, such as smoking, as well as underlying diseases, than women [11]. Moreover, as mentioned in Huang’s report [1], this may be associated with the risk factors that are related to men’s occupations, such as the exposure in Huanan wet market. Furthermore, based on our results, older people (mean age = 58.42) are more prone to COVID-19, which could be related to an increase in the frequency of common comorbidities [32]. As our body’s immunity weakens with age, elderly patients are more likely to have serious ailments or even die [11]. Hospitalization and further ICU admission is prevalent among sever cases of COVID-19, especially the ones with underlying health conditions and elderly patients [123]. Based on a CDC report, 8 death cases out of every 10 death cases occurring in the USA are old individuals (i.e., 65 years old and above) [124]. Additionally, it has been reported that patients with comorbid conditions, such as diabetes, hypertension, cardiovascular disease, obesity, and chronic lung diseases, are more likely to have a worse prognosis and develop severe pneumonia and further ARDS [125].

Our study provides satisfactory evidence for the risk factors in severely-ill COVID-19 patients, such as characteristics of the patients (gender, age), comorbidity (hypertension, chronic kidney disease, diabetes, cardiovascular and cerebrovascular diseases, and coronary heart disease), and signs and symptoms (fever, diarrhea, fatigue or myalgia, and dyspnea) [25]. Furthermore, we have discovered that underlying diseases such as diabetes, hypertension, cardiovascular disease, and chronic kidney disease can be considered as risk factors for the progression of this disease, which is confirmed by the analytical results of our study. Moreover, the most common clinical symptoms of COVID-19 in infected patients, including fever, diarrhea, fatigue or myalgia, and dyspnea, have been assessed in this paper. Dyspnea or shortness of breath indicates lack of oxygen and poor lung function. Therefore, if the patient is an elderly male with underlying diseases, he is more likely to have severe disorders or even face death [11].

Neurological complications can also occur as a result of COVID-19; however, they are very rare (especially in contrast to respiratory complications). According to recent reports, neurological manifestations of COVID-19 include headache, vomiting, nausea, dizziness, hyposmia, hypogeusia, and impaired consciousness [12, 78]. Although the exact neuroinvasive route of SARS-CoV-2 has not yet been established, two hypothetical routes have been suggested, i.e., (1) neural dissemination through the olfactory nerve and the cribriform plate, and (2) hematogenous dissemination through cerebral circulation [126, 127]. According to these possible routes, neural invasion of SARS-CoV-2 seems possible. Additionally, it has been proved that there is a close relationship between neurological disorders and COVID-19; however, a big question that arises in this regard is whether this relationship is causal or not (i.e., does one of them affects the mortality rate or the incident of the other one or not?). The answer to this question has not yet been given; however, there is some evidence showing that SARS-CoV-2 infection can affect the clinical spectrum of neurological disorders [128].

According to the selected papers assessed in the current study, fever with 79.26% (95% CI: 74.98–83.26; I^2^ = 97.35%) is the most common symptom, while hypertension with 28.30% (95% CI: 23.66–33.18; I^2^ = 99.58%) can be considered as the most prevalent comorbidity. When comparing our results with those of other recent meta-analysis studies, the study conducted by Rodriguez-Morales et al. [129] confirms our findings. These authors found that fever with 88.7% (95% CI 84.5–92.9) was the most prevalent symptom, and hypertension with 18.6% (95% CI 8.1–29.0) was the most common comorbidity. However, the number of selected studies in our meta-analysis was larger than the size of their dataset; hence, our findings are more reliable.

Our findings are also in line with the results of the study by Jing Yang et al. [14], in which fever with 91.3% (95% CI: 86–97) was the most prevalent symptom, and hypertension with 21.1% (95% CI: 13.0–27.2) was the most common comorbidity. In addition, in a study by Emami et al. [130], hypertension was reported as the most prevalent comorbidity with 16.37% (95%CI: 10.15–23.65). Moreover, the prevalence of diabetes mellitus and hypertension in China were 10.9% [131] and 23.2% [132] in adults, respectively.

Based on the abovementioned studies and our results, individuals with cardiovascular diseases, hypertension, chronic kidney disease, and diabetes should receive serious attention for treatments and vaccinations useful for SARS-CoV-2 in the future. Due to limited evidence, more comprehensive studies are needed to demonstrate this correlation [14].

Unfortunately, no anti-virus vaccines or medicines are yet known for the treatment of COVID-19-related diseases. At the present time, clinical management, including infection prevention, supportive care, and control measures, such as mechanical ventilation and supplementary oxygen, are available. Antibiotics are not useful in curing COVID-19 patients; however, they can be employed in the case of a secondary bacterial infection.

#### Favilavir

Favilavir is an antiviral drug approved in 2014 in Japan as a treatment for influenza. It is currently in use in some countries as a treatment for COVID-19 [133]. Another anti-virus drug which can have useful effects for COVID-19 is called Remdesivir. It has demonstrated efficacy by resisting two viruses that are similar to SARS-CoV2, i.e., MERS-CoV and SARS-CoV in animals [134, 135].

#### Remdesivir

Remdesivir was employed to cure the first case of COVID-19 infection in the US, resulting in enhancing the conditions of the patient after only one day of using Remdesivir [136]. Based on the related investigations, Remdesivir improved severe lung pathology, reduced lung viral loads, and ameliorated pulmonary function. In addition, chloroquine was suggested in the recommendations for the treatment and prevention of COVID-19 pneumonia [137]. Nevertheless, the optimal dosage of chloroquine for treating SARS-CoV-2 needs to be evaluated in future trials [138]. Hydroxychloroquine is an analog of chloroquine which is used to reduce drug-drug interactions [139]. There is no approved data about the use of corticosteroids for the treatment of SARS-CoV2 [140, 141]. Overall, there is no specific reason yet to expect that patients with COVID- 19 infection will improve by using corticosteroids; hence, these treatments may have harmful effects [142, 143].

A recent clinical trial by [144] suggests that Remdesivir is a therapeutic option for COVID-19 patients who do not receive invasive ventilation. Based on the clinical and experimental results, clinical trials for evaluating the efficacy of Remdesivir in COVID-19 patients have recently been initiated in the USA, China [145, 146], Hong Kong, the Republic of Korea, Singapore [147], Taiwan [148, 149], and France [150, 151].

#### Lopinavir/Ritonavir

Moreover, based on the investigations, treatment with lopinavir/ritonavir does not decrease the duration of viral RNA or viral RNA loads [11]. In addition, the number of patients treated with lopinavir/ritonavir who developed serious complications, such as secondary infections and acute kidney injury, or who required invasive or noninvasive mechanical ventilation due to respiratory failure, was lower than the number of patients who did not receive lopinavir/ritonavir treatment [11]. These observations need additional studies to find out whether treatment with lopinavir/ritonavir is able to decrease some complications in COVID-19 patients [11, 152].

On February 24, 2020, Moderna Company stated that the company’s experimental mRNA COVID-19 vaccine, which is known as mRNA-1273, was ready to be tested on human subjects. It is a considerably fast progress to produce an initial vaccine just a few weeks after determining the SARS-CoV-2 genetic sequence. However, the clinical trial of the immunogenicity and safety of mRNA-1273 in the treatment of COVID-19 needs to be evaluated more extensively (clinicaltrials.gov Identifier: NCT04283461). Additionally, a new oral SARS-CoV-2 vaccine has been successfully produced at Tianjin University, which employs food-grade safe *Saccharomyces cerevisiae* as a transporter and targets the S protein. There are 18 biotechnology universities and companies in China studying potential SARS-CoV-2 vaccines [143].

Current diagnostic tests for coronaviruses include real-time RT-PCR (rRT-PCR), reverse transcription loop-mediated isothermal amplification (RT-LAMP), and reverse-transcription polymerase chain reaction (RT-PCR) [153]. RT-LAMP has the same sensitivity as rRT-PCR; however, it is highly particular and is employed to detect MERS-CoV [154, 155]. Based on the current diagnosis represented by the laboratory examinations of the Chinese National Health Commission, oropharyngeal and nasopharyngeal swab tests have become a standard evaluation for the diagnosis of COVID-19 infection. A large portion of the vaccines being produced for coronaviruses target the spike glycoprotein or the S protein [156]. However, vaccine development takes too long, and no vaccines are available at the time of this pandemic [143].

In addition, COVID-19 prevents access to health care for the most vulnerable individuals due to health inequalities [157]. Although structural racism has been shown to affect the distribution of health determinants and risk factors in society, but due to the lack of understanding on the exact influence of these variables on social health, appropriate action is not given by the health system [158, 159]. Focus on addressing both unsupplied social needs (i.e. the immediate social conditions that put the greatest pressure on individuals) and social risk factors (adverse social conditions such as those associated with poor health) can help us solve this problem with a more holistic point of view [158].

Inopportunely, the unfortunate consequences of structural racism, which has been a problem since the days of the transatlantic slave trade, have continued to this day and include a more high poverty rate, an imperfect public school system, police brutality, unsafe neighborhoods and, above all, or, higher rate of chronic health conditions, obesity, and infant and maternal mortality among black American populations [160]. In compatibility with these unfortunate facts, the US national COVID-19 hospitalization rate was 4.6 times higher in Latins and the death rate was 2.1 times higher in black Americans than in whites [161]. Furthermore, it was reported that pregnancy-related complications and further death were three to four times higher in black women than in white women and were also twice as likely to die from heart disease [162–164]. Consequently, a powerful multidisciplinary and coordinated effort must be given by whoever is responsible, including politicians, policy makers, public health professionals, educators and local community leaders, to provide, systematically and actively improve health equity [161].

This data is not incredible or new. Because of these statistics, the chance of death due to pregnancy-related outcomes in black women is three to four times higher than in white women. The probability of death from heart disease in black women is also twice as high as in white women [161, 163, 165, 166]. The Covid-19 epidemic shows the relationship between social risk factors, health and structural racism. The prevalence of diabetes among black Americans has been reported 1.7 times greater than in whites [166]. The prevalence of influenza-related hospitalization in black Americans has been shown to be 1.8 times that of white Americans in the past 10 flu seasons [161]. It has been hypothesized that the increased exposure to COVID-19 among the black American population is due to the higher rate of service occupations and the greater possibility of living in more crowded and dense cities [167–169]. In the current economy, people of color are more likely to lose their jobs due to the potential consequences of the prevalence of COVID-19: 16.8% of black workers and 17.6% of Hispanic workers are at risk of losing their jobs compared to approximately 12.5% of non-Hispanic whites [161]. However, more research is needed for the structural factors leading to these statistics [169].

Early diagnosis and isolation of the patients constitute a key mechanism in preventing the spread of communicable diseases; however, as SARS-Cov-2 has other transmission routes (e.g., fomite transmission), it is even harder to control its rapid global spread [170]. Recently, it has been reported that SARS-CoV-2 is also detectable in saliva, urine, stool, and gastric mucosa [11, 171, 172], which can be related to its high transmission rate. In conclusion, the best protection against infection in the population is improving personal protection and managing hygiene at the community level [170]. According to an experience in Hangzhou City, China, centralized medical observation points (medical observation of close contacts) can also be helpful in controlling the COVID-19 outbreak [173].

In summary, the results of the current review indicate that in COVID-19 patients, diabetes, hypertension, cardiovascular disease, and chronic kidney disease are the most common comorbidities, while fever, diarrhea, fatigue or myalgia, and dyspnea may be considered as the most prevalent symptoms among COVID-19 patients. Since SARS-CoV-2 has a long incubation period and the infected individuals can transmit this contagious virus even without manifesting obvious symptoms, it is highly recommended that sick patients who have underlying disorders, especially in epidemic areas, refrain from coming into contact with other people in the society. Additionally, other strategies, such as avoiding gathering in public areas and going to epidemic places, should be considered as guidelines. During the current COVID-19 outbreak, the reported statistics, related to overall mortality, incubation time, and in particular the risk factors, imply the need for serious decisions for future prevention and therapeutic actions [130].

## Conclusions

This systematic review and meta-analysis summarized the current evidence on the prevalence of mortality and potential risk factors in patients with COVID-19. Based on the results of this meta-analysis, COVID-19 is closely associated with underlying diseases. According to our results, the most frequent comorbidities among hospitalized COVID-19 patients were hypertension, diabetes, cardiovascular disease, and chronic kidney disease, respectively. The results also show that old men with underlying diseases are at a higher risk of becoming infected with COVID-19. Therefore, given the high risk of death in patients with COVID-19 infection, special care and significant attention are needed in COVID-19 patients with associated conditions. Moreover, future studies are necessary to identify efficient treatments for severe COVID-19 patients who suffer from diabetes, hypertension, chronic kidney disease, or cardiovascular diseases.

## Supporting information

S1 Checklist PRISMA 2009 checklist. (PDF)

S1 Data (SAV)

S1 File (DOCX)

## Abbreviations

WHO: World Health Organization
ICU: intensive care unit
ACE2: angiotensin-converting enzyme 2
No: Number

## References

[1] Huang C., et al, Clinical features of patients infected with 2019 novel coronavirus in Wuhan, China. The lancet, 2020 395(10223): p. 497–506. 10.1016/S0140-6736(20)30183-5 PMC7159299 31986264

[2] Huang Y., et al, Does comorbidity increase the risk of patients with COVID-19: evidence from meta-analysis. Aging, 2020 12(7): p. 6049–6057. 10.18632/aging.103000 32267833 PMC7185114

[3] Organization W.H., WHO announces COVID-19 outbreak a pandemic. WHO, Geneva, Switzerland, 2020.

[4] Kreutz R., et al, Hypertension, the renin–angiotensin system, and the risk of lower respiratory tract infections and lung injury: implications for COVID-19European Society of Hypertension COVID-19 Task Force Review of Evidence. Cardiovascular Research, 2020. 10.1093/cvr/cvab224 PMC8344701 34269396

[5] de Abajo F.J., et al, Use of renin–angiotensin–aldosterone system inhibitors and risk of COVID-19 requiring admission to hospital: a case-population study. The Lancet, 2020 10.1016/S0140-6736(20)31030-8 32416785 PMC7255214

[6] Kelvin D.J. and Rubino S., Fear of the novel coronavirus. The Journal of Infection in Developing Countries, 2020 14(01): p. 1–2. 10.3855/jidc.12496 32088678

[7] Carlos W.G., et al, Novel wuhan (2019-nCoV) coronavirus. American journal of respiratory and critical care medicine, 2020 201(4): p. P7–P8. 10.1164/rccm.2014P7 32004066

[8] Paules C.I., Marston H.D., and Fauci A.S., Coronavirus infections—more than just the common cold. Jama, 2020 323(8): p. 707–708. 10.1001/jama.2020.0757 31971553

[9] Livingston E., Bucher K., and Rekito A., Coronavirus Disease 2019 and Influenza 2019–2020. Jama, 2020 323(12): p. 1122–1122. 10.1001/jama.2020.2633 32207769

[10] Liu W., et al, Analysis of factors associated with disease outcomes in hospitalized patients with 2019 novel coronavirus disease. Chinese medical journal, 2020 10.1097/CM9.0000000000000775 32118640 PMC7147279

[11] Guan W.-j., et al, Clinical characteristics of coronavirus disease 2019 in China. New England journal of medicine, 2020 382(18): p. 1708–1720. 10.1056/NEJMoa2002032 PMC7092819 32109013

[12] Wang D., et al, Clinical characteristics of 138 hospitalized patients with 2019 novel coronavirus–infected pneumonia in Wuhan, China. Jama, 2020 323(11): p. 1061–1069. 10.1001/jama.2020.1585 PMC7042881 32031570

[13] Lai C.-C., et al, Global epidemiology of coronavirus disease 2019: disease incidence, daily cumulative index, mortality, and their association with country healthcare resources and economic status. International Journal of Antimicrobial Agents, 2020: p. 105946 10.1016/j.ijantimicag.2020.105946 32199877 PMC7156123

[14] Yang J., et al, Prevalence of comorbidities in the novel Wuhan coronavirus (COVID-19) infection: a systematic review and meta-analysis. International Journal of Infectious Diseases, 2020.

[15] Freeman M.F. and Tukey J.W., Transformations related to the angular and the square root. The Annals of Mathematical Statistics, 1950: p. 607–611.

[16] Wells G., The Newcastle-Ottawa Scale (NOS) for assessing the quality of nonrandomised studies in meta-analysis. http://www.ohri.ca/programs/clinical_epidemiology.oxford.htm, 2004.

[17] Liberati A., et al, The PRISMA statement for reporting systematic reviews and meta-analyses of studies that evaluate health care interventions: explanation and elaboration. Annals of internal medicine, 2009 151(4): p. W-65–W-94. 19622512 10.7326/0003-4819-151-4-200908180-00136

[18] Zhou F., et al, Clinical course and risk factors for mortality of adult inpatients with COVID-19 in Wuhan, China: a retrospective cohort study. The lancet, 2020 10.1016/S0140-6736(20)30566-3 32171076 PMC7270627

[19] Du Y., et al, Clinical features of 85 fatal cases of COVID-19 from Wuhan: a retrospective observational study. American journal of respiratory and critical care medicine, 2020(ja). 10.1164/rccm.202003-0543OC 32242738 PMC7258652

[20] Zhang J.-j., et al, Clinical characteristics of 140 patients infected with SARS‐CoV‐2 in Wuhan, China. Allergy, 2020 10.1111/all.14238 32077115

[21] Guo W., et al, Diabetes is a risk factor for the progression and prognosis of COVID‐19. Diabetes/metabolism research and reviews, 2020 10.1002/dmrr.3319 32233013 PMC7228407

[22] Guan W.-j., et al, Comorbidity and its impact on 1590 patients with Covid-19 in China: A Nationwide Analysis. European Respiratory Journal, 2020 10.1183/13993003.00547-2020 32217650 PMC7098485

[23] Wu C., et al, Risk factors associated with acute respiratory distress syndrome and death in patients with coronavirus disease 2019 pneumonia in Wuhan, China. JAMA internal medicine, 2020 10.1001/jamainternmed.2020.0994 PMC7070509 32167524

[24] Wang L., et al, Coronavirus Disease 2019 in elderly patients: characteristics and prognostic factors based on 4-week follow-up. Journal of Infection, 2020 10.1016/j.jinf.2020.03.019 32240670 PMC7118526

[25] Li X., et al, Clinical characteristics of 25 death cases with COVID-19: a retrospective review of medical records in a single medical center, Wuhan, China. International Journal of Infectious Diseases, 2020. 10.1016/j.ijid.2020.03.053 PMC7128884 32251805

[26] Chow N., et al, Preliminary estimates of the prevalence of selected underlying health conditions among patients with coronavirus disease 2019—United States, February 12–March 28, 2020. 2020. 10.15585/mmwr.mm6913e2 PMC7119513 32240123

[27] Garg S., Hospitalization rates and characteristics of patients hospitalized with laboratory-confirmed coronavirus disease 2019—COVID-NET, 14 States, March 1–30, 2020. MMWR. Morbidity and Mortality Weekly Report, 2020 69 10.15585/mmwr.mm6915e3 32298251 PMC7755063

[28] Wang X., et al, Clinical characteristics of non-critically ill patients with novel coronavirus infection (COVID-19) in a Fangcang Hospital. Clinical Microbiology and Infection, 2020. 10.1016/j.cmi.2020.03.032 PMC7195539 32251842

[29] Chen N., et al, Epidemiological and clinical characteristics of 99 cases of 2019 novel coronavirus pneumonia in Wuhan, China: a descriptive study. The Lancet, 2020 395(10223): p. 507–513. 10.1016/S0140-6736(20)30211-7 32007143 PMC7135076

[30] Li J., et al, Epidemiological and Clinical Characteristics of 17 Hospitalized Patients with 2019 Novel Coronavirus Infections Outside Wuhan, China. medRxiv, 2020.

[31] Xu X.-W., et al, Clinical findings in a group of patients infected with the 2019 novel coronavirus (SARS-Cov-2) outside of Wuhan, China: retrospective case series. bmj, 2020 368. 10.1136/bmj.m606 PMC7224340 32075786

[32] Wu J., et al, Clinical Characteristics of Imported Cases of Coronavirus Disease 2019 (COVID-19) in Jiangsu Province: A Multicenter Descriptive Study. Clinical Infectious Diseases, 2020 10.1093/cid/ciaa199 32109279 PMC7108195

[33] Liu K., et al, Clinical characteristics of novel coronavirus cases in tertiary hospitals in Hubei Province. Chinese medical journal, 2020 10.1097/CM9.0000000000000744 32044814 PMC7147277

[34] Liu J., et al, Neutrophil-to-lymphocyte ratio predicts severe illness patients with 2019 novel coronavirus in the early stage. MedRxiv, 2020. 10.1186/s12967-020-02374-0 PMC7237880 32434518

[35] Yang X., et al, Clinical course and outcomes of critically ill patients with SARS-CoV-2 pneumonia in Wuhan, China: a single-centered, retrospective, observational study. The Lancet Respiratory Medicine, 2020 10.1016/S2213-2600(20)30079-5 32105632 PMC7102538

[36] Guo T., et al, Cardiovascular implications of fatal outcomes of patients with coronavirus disease 2019 (COVID-19). JAMA cardiology, 2020. 10.1001/jamacardio.2020.1017 PMC7101506 32219356

[37] Onder G., Rezza G., and Brusaferro S., Case-fatality rate and characteristics of patients dying in relation to COVID-19 in Italy. Jama, 2020 10.1001/jama.2020.4683 32203977

[38] Liu Y., et al, Clinical and biochemical indexes from 2019-nCoV infected patients linked to viral loads and lung injury. Science China Life Sciences, 2020 63(3): p. 364–374. 10.1007/s11427-020-1643-8 32048163 PMC7088566

[39] Zhang M.Q., et al, [Clinical features of 2019 novel coronavirus pneumonia in the early stage from a fever clinic in Beijing]. Zhonghua Jie He He Hu Xi Za Zhi, 2020 43(0): p. E013 10.3760/cma.j.issn.1001-0939.2020.0013 32061066

[40] Surveillances V., The epidemiological characteristics of an outbreak of 2019 novel coronavirus diseases (COVID-19)—China, 2020. China CDC Weekly, 2020 2(8): p. 113–122. PMC8392929 34594836

[41] Cheng Y., et al, Kidney disease is associated with in-hospital death of patients with COVID-19. Kidney International, 2020. 10.1016/j.kint.2020.03.005 PMC7110296 32247631

[42] Li Z., et al, Caution on kidney dysfunctions of COVID-19 patients. 2020.

[43] Chen T., et al, Clinical characteristics of 113 deceased patients with coronavirus disease 2019: retrospective study. Bmj, 2020 368 10.1136/bmj.m1091 32217556 PMC7190011

[44] Zhang B., et al, Clinical characteristics of 82 death cases with COVID-19. medRxiv, 2020.

[45] Zhang H., et al, Identification of kidney transplant recipients with coronavirus disease 2019. European Urology, 2020 10.1016/j.eururo.2020.03.030 32249089 PMC7270372

[46] Shi S., et al, Association of cardiac injury with mortality in hospitalized patients with COVID-19 in Wuhan, China. JAMA cardiology, 2020 10.1001/jamacardio.2020.0950 PMC7097841 32211816

[47] Wang L., et al, Coronavirus disease 19 infection does not result in acute kidney injury: an analysis of 116 hospitalized patients from Wuhan, China. American journal of nephrology, 2020 51(5): p. 343–348. 10.1159/000507471 32229732 PMC7179524

[48] Arentz M., et al, Characteristics and outcomes of 21 critically ill patients with COVID-19 in Washington State. Jama, 2020 323(16): p. 1612–1614. 10.1001/jama.2020.4326 32191259 PMC7082763

[49] Chen T., et al, Clinical characteristics and outcomes of older patients with coronavirus disease 2019 (COVID-19) in Wuhan, China (2019): a single-centered, retrospective study. The Journals of Gerontology: Series A, 2020 10.1093/gerona/glaa089 32279081 PMC7184388

[50] Qian G.-Q., et al, Epidemiologic and Clinical Characteristics of 91 Hospitalized Patients with COVID-19 in Zhejiang, China: A retrospective, multi-centre case series. QJM: An International Journal of Medicine, 2020 10.1093/qjmed/hcaa089 32181807 PMC7184349

[51] Qin C., et al, Dysregulation of immune response in patients with COVID-19 in Wuhan, China. Clinical Infectious Diseases, 2020 10.1093/cid/ciaa248 32161940 PMC7108125

[52] Wang Z., et al, Clinical Features of 69 Cases with Coronavirus Disease 2019 in Wuhan, China. Clin Infect Dis, 2020 10.1093/cid/ciaa272 32176772 PMC7184452

[53] Zhang G., et al, Clinical features and short-term outcomes of 221 patients with COVID-19 in Wuhan, China. Journal of Clinical Virology, 2020: p. 104364 10.1016/j.jcv.2020.104364 PMC7194884 32311650

[54] Xu X., et al, Imaging and clinical features of patients with 2019 novel coronavirus SARS-CoV-2. European journal of nuclear medicine and molecular imaging, 2020: p. 1–6. 10.1007/s00259-020-04735-9 32107577 PMC7080117

[55] Du R.-H., et al, Hospitalization and Critical Care of 109 Decedents with COVID-19 Pneumonia in Wuhan, China. Annals of the American Thoracic Society, 2020(ja). 10.1513/AnnalsATS.202003-225OC 32255382 PMC7328178

[56] Cao J., et al, Clinical Features and Short-term Outcomes of 102 Patients with Corona Virus Disease 2019 in Wuhan, China. Clinical Infectious Diseases, 2020. 10.1093/cid/ciaa243 PMC7184479 32239127

[57] Grasselli G., et al, Baseline characteristics and outcomes of 1591 patients infected with SARS-CoV-2 admitted to ICUs of the Lombardy Region, Italy. Jama, 2020 10.1001/jama.2020.5394 PMC7136855 32250385

[58] Richardson S., et al, Presenting Characteristics, Comorbidities, and Outcomes Among 5700 Patients Hospitalized With COVID-19 in the New York City Area. JAMA, 2020. 10.1001/jama.2020.6775 PMC7177629 32320003

[59] He X.W., et al, [Impact of complicated myocardial injury on the clinical outcome of severe or critically ill COVID-19 patients]. Zhonghua Xin Xue Guan Bing Za Zhi, 2020 48(0): p. E011. 10.3760/cma.j.cn112148-20200228-00137 32171190

[60] Fu L., et al, Influence factors of death risk among COVID-19 patients in Wuhan, China: a hospital-based case-cohort study. medRxiv, 2020: p. 2020.03.13.20035329.

[61] Han Y., et al, Lactate dehydrogenase, a Risk Factor of Severe COVID-19 Patients. medRxiv, 2020 10.18632/aging.103372 32633729 PMC7343511

[62] Zhang F., et al, Myocardial injury is associated with in-hospital mortality of confirmed or suspected COVID-19 in Wuhan, China: A single center retrospective cohort study. MedRxiv, 2020.

[63] Hu L., et al, Risk factors associated with clinical outcomes in 323 COVID-19 patients in Wuhan, China. Medrxiv, 2020 10.1093/cid/ciaa539 32361738 PMC7197620

[64] Ji D., et al, Clinical Characteristics Predicting Progression of COVID-19. 2020.

[65] Cai Q., et al, 2019-nCoV Pneumonia in a Normal Work Infectious Diseases Hospital Besides Hubei Province, China. 2020.

[66] Lu H., et al, A descriptive study of the impact of diseases control and prevention on the epidemics dynamics and clinical features of SARS-CoV-2 outbreak in Shanghai, lessons learned for metropolis epidemics prevention. medRxiv, 2020.

[67] Wang G., et al, Epidemiological and Clinical Features of Corona Virus Disease 2019 (COVID-19) in Changsha, China. China (3/1/2020), 2020.

[68] Ma K.-L., et al, COVID-19 myocarditis and severity factors: an adult cohort study. medRxiv, 2020.

[69] Liu Y., et al, Clinical features and progression of acute respiratory distress syndrome in coronavirus disease 2019 MedRxiv, 2020.

[70] Li X., et al, Risk factors for severity and mortality in adult COVID-19 inpatients in Wuhan. Journal of Allergy and Clinical Immunology, 2020 10.1016/j.jaci.2020.04.006 32294485 PMC7152876

[71] Xu K., et al, Factors associated with prolonged viral RNA shedding in patients with COVID-19. Clinical Infectious Diseases, 2020. 10.1093/cid/ciaa351 PMC7184421 32271376

[72] Yuan M., et al, Association of radiologic findings with mortality of patients infected with 2019 novel coronavirus in Wuhan, China. PLoS One, 2020 15(3): p. e0230548 10.1371/journal.pone.0230548 PMC7082074 32191764

[73] Lian J., et al, Analysis of Epidemiological and Clinical features in older patients with Corona Virus Disease 2019 (COVID-19) out of Wuhan. Clinical Infectious Diseases, 2020. 10.1093/cid/ciaa242 PMC7184356 32211844

[74] Escalera-Antezana J.P., et al, Clinical features of cases and a cluster of Coronavirus Disease 2019 (COVID-19) in Bolivia imported from Italy and Spain. Travel Medicine and Infectious Disease, 2020: p. 101653 10.1016/j.tmaid.2020.101653 32247926 PMC7129170

[75] Zheng F., et al, Clinical characteristics of 161 cases of corona virus disease 2019 (COVID-19) in Changsha. Eur Rev Med Pharmacol Sci, 2020 24(6): p. 3404–3410. 10.26355/eurrev_202003_20711 32271459

[76] Wan S., et al, Clinical Features and Treatment of COVID‐19 Patients in Northeast Chongqing. Journal of medical virology, 2020 10.1002/jmv.25783 32198776 PMC7228368

[77] Zhao S., et al, Anesthetic management of patients with suspected or confirmed 2019 novel coronavirus infection during emergency procedures. J Cardiothorac Vasc Anesth, 2020 28: p. 28. 10.1053/j.jvca.2020.02.039 PMC7102598 32178954

[78] Mao L., et al, Neurologic manifestations of hospitalized patients with coronavirus disease 2019 in Wuhan, China. JAMA neurology, 2020 10.1001/jamaneurol.2020.1127 32275288 PMC7149362

[79] Deng Q., et al, Suspected myocardial injury in patients with COVID-19: Evidence from front-line clinical observation in Wuhan, China. International Journal of Cardiology, 2020 10.1016/j.ijcard.2020.03.087 PMC7141178 32291207

[80] Shao F., et al, In-hospital cardiac arrest outcomes among patients with COVID-19 pneumonia in Wuhan, China. Resuscitation, 2020 10.1016/j.resuscitation.2020.04.005 32283117 PMC7151543

[81] Tu W.-J., et al, Clinicolaboratory study of 25 fatal cases of COVID-19 in Wuhan. Intensive care medicine, 2020: p. 1–4. 10.1007/s00134-020-06023-4 32253448 PMC7131987

[82] Young B.E., et al, Epidemiologic features and clinical course of patients infected with SARS-CoV-2 in Singapore. Jama, 2020 323(15): p. 1488–1494. 10.1001/jama.2020.3204 32125362 PMC7054855

[83] Park S., et al, Analysis on 54 Mortality Cases of Coronavirus Disease 2019 in the Republic of Korea from January 19 to March 10, 2020. J Korean Med Sci, 2020 35(12): p. e132 10.3346/jkms.2020.35.e132 32233161 PMC7105509

[84] Yang F., et al, Analysis of 92 deceased patients with COVID‐19. Journal of Medical Virology, 2020. 10.1002/jmv.25891 PMC7262332 32293741

[85] Meng Y., et al, Sex-specific clinical characteristics and prognosis of coronavirus disease-19 infection in Wuhan, China: A retrospective study of 168 severe patients. PLoS pathogens, 2020 16(4): p. e1008520 10.1371/journal.ppat.1008520 32343745 PMC7209966

[86] Li Y.-K., et al, Clinical and transmission characteristics of Covid-19—a retrospective study of 25 cases from a single thoracic surgery department. Current Medical Science, 2020: p. 1–6. 10.1007/s11596-020-2140-1 32232652 PMC7104422

[87] Lovell N., et al, Characteristics, symptom management and outcomes of 101 patients with COVID-19 referred for hospital palliative care. Journal of Pain and Symptom Management, 2020. 10.1016/j.jpainsymman.2020.04.015 PMC7169932 32325167

[88] Liu C., et al, Clinical features and multidisciplinary treatment outcome of COVID-19 pneumonia: A report of three cases. Journal of the Formosan Medical Association, 2020 10.1016/j.jfma.2020.04.008 32317205 PMC7161490

[89] Yang W., et al, Clinical characteristics and imaging manifestations of the 2019 novel coronavirus disease (COVID-19): A multi-center study in Wenzhou city, Zhejiang, China. Journal of Infection, 2020 10.1016/j.jinf.2020.02.016 32112884 PMC7102539

[90] Shi H., et al, Radiological findings from 81 patients with COVID-19 pneumonia in Wuhan, China: a descriptive study. The Lancet Infectious Diseases, 2020 10.1016/S1473-3099(20)30086-4 32105637 PMC7159053

[91] Choe Y.J., Coronavirus disease-19: The First 7,755 Cases in the Republic of Korea. medRxiv, 2020. 10.24171/j.phrp.2020.11.2.05 PMC7104685 32257774

[92] Yao Q., et al, Retrospective study of risk factors for severe SARS-Cov-2 infections in hospitalized adult patients. Polish archives of internal medicine, 2020 10.20452/pamw.15312 32329978

[93] Feng Y., et al, COVID-19 with Different Severity: A Multi-center Study of Clinical Features. American Journal of Respiratory and Critical Care Medicine, 2020(ja). 10.1164/rccm.202002-0445OC PMC7258639 32275452

[94] Mo P., et al, Clinical characteristics of refractory COVID-19 pneumonia in Wuhan, China. Clinical Infectious Diseases, 2020 10.1093/cid/ciaa270 32173725 PMC7184444

[95] Shi Y., et al, Host susceptibility to severe COVID-19 and establishment of a host risk score: findings of 487 cases outside Wuhan. Critical Care, 2020 24(1): p. 1–4. 10.1186/s13054-019-2683-3 32188484 PMC7081524

[96] McMichael T.M., et al, Epidemiology of Covid-19 in a long-term care facility in King County, Washington. New England Journal of Medicine, 2020 382(21): p. 2005–2011. 10.1056/NEJMoa2005412 PMC7121761 32220208

[97] Fabio C., et al, Early predictors of clinical outcomes of COVID-19 outbreak in Milan, Italy. Clinical Immunology, 2020: p. 108509 10.1016/j.clim.2020.108509 PMC7289745 32535188

[98] Duanmu Y., et al, Characteristics of Emergency Department Patients With COVID‐19 at a Single Site in Northern California: Clinical Observations and Public Health Implications. Academic Emergency Medicine, 2020. 10.1111/acem.14003 PMC7267565 32344458

[99] Inciardi R.M., et al, Characteristics and outcomes of patients hospitalized for COVID-19 and cardiac disease in Northern Italy. European heart journal, 2020 41(19): p. 1821–1829. 10.1093/eurheartj/ehaa388 32383763 PMC7239204

[100] Buckner F.S., et al, Clinical Features and Outcomes of 105 Hospitalized patients with COVID-19 in Seattle, Washington. Clinical Infectious Diseases, 2020 10.1093/cid/ciaa632 32444880 PMC7314181

[101] Halvatsiotis P., et al, Demographic and clinical features of critically ill patients with COVID-19 in Greece: The burden of diabetes and obesity. Diabetes research and clinical practice, 2020 166: p. 108331 10.1016/j.diabres.2020.108331 32682810 PMC7366091

[102] Colombi D., et al, Well-aerated lung on admitting chest CT to predict adverse outcome in COVID-19 pneumonia. Radiology, 2020: p. 201433 10.1148/radiol.2020201433 32301647 PMC7233411

[103] Marcello R.K., et al, Characteristics and Outcomes of COVID-19 Patients in New York City’s Public Hospital System. medRxiv, 2020. 10.1371/journal.pone.0243027 PMC7745980 33332356

[104] Li K., et al, The clinical and chest CT features associated with severe and critical COVID-19 pneumonia. Investigative radiology, 2020 10.1097/RLI.0000000000000672 32118615 PMC7147273

[105] Wang K., et al, Clinical and laboratory predictors of in-hospital mortality in patients with COVID-19: a cohort study in Wuhan, China. Clinical infectious diseases, 2020. 10.1093/cid/ciaa538 PMC7197616 32361723

[106] Liang W., et al, Development and validation of a clinical risk score to predict the occurrence of critical illness in hospitalized patients with COVID-19. JAMA Internal Medicine, 2020 10.1001/jamainternmed.2020.2033 32396163 PMC7218676

[107] Mancia G., et al, Renin–angiotensin–aldosterone system blockers and the risk of Covid-19. New England Journal of Medicine, 2020 10.1056/NEJMoa2006923 32356627 PMC7206933

[108] Mehta N., et al, Association of use of angiotensin-converting enzyme inhibitors and angiotensin II receptor blockers with testing positive for coronavirus disease 2019 (COVID-19). JAMA cardiology, 2020. 10.1001/jamacardio.2020.1855 PMC7201375 32936273

[109] Bean D., et al, ACE-inhibitors and Angiotensin-2 Receptor Blockers are not associated with severe SARS-COVID19 infection in a multi-site UK acute Hospital Trust. MedRxiv, 2020. 10.1002/ejhf.1924 PMC7301045 32485082

[110] Caraballo C., et al, COVID-19 Infections and Outcomes in a Live Registry of Heart Failure Patients Across an Integrated Health Care System. medRxiv, 2020. 10.1371/journal.pone.0238829 PMC7526909 32997657

[111] De Spiegeleer A., et al, The effects of ARBs, ACEIs and statins on clinical outcomes of COVID-19 infection among nursing home residents. medRxiv, 2020 10.1016/j.jamda.2020.06.018 32674818 PMC7294267

[112] Ebinger J.E., et al, Pre-Existing Characteristics Associated with Covid-19 Illness Severity. medRxiv, 2020 10.1371/journal.pone.0236240 32702044 PMC7377468

[113] Khawaja A.P., et al, Associations with covid-19 hospitalisation amongst 406,793 adults: the UK Biobank prospective cohort study. medRxiv, 2020.

[114] Raisi-Estabragh Z., et al, NON-WHITE ETHNICITY, MALE SEX, AND HIGHER BODY MASS INDEX, BUT NOT MEDICATIONS ACTING ON THE RENIN-ANGIOTENSIN SYSTEM ARE ASSOCIATED WITH CORONAVIRUS DISEASE 2019 (COVID-19) HOSPITALISATION: REVIEW OF THE FIRST 669 CASES FROM THE UK BIOBANK. medRxiv, 2020.

[115] Ghebreyesus T.A., WHO Director-General’s opening remarks at the media briefing on COVID-19-11 March 2020. World Health Organization https://www.who.int/dg/speeches/detail/who-director-general-s-opening-remarks-atthe-media-briefing-on-covid-19—11-march-2020, 2020.

[116] Li J. and Xu G., Lessons from the Experience in Wuhan to Reduce Risk of COVID-19 Infection in Patients Undergoing Long-Term Hemodialysis. Clinical Journal of the American Society of Nephrology, 2020 10.2215/CJN.03420320 32241778 PMC7269214

[117] Chan J.F.-W., et al, A familial cluster of pneumonia associated with the 2019 novel coronavirus indicating person-to-person transmission: a study of a family cluster. The Lancet, 2020 395(10223): p. 514–523. 10.1016/S0140-6736(20)30154-9 31986261 PMC7159286

[118] Guan W., et al, China Medical Treatment Expert Group for Covid-19. Clinical characteristics of coronavirus disease, 2019.

[119] Tipnis S., Hooper NM, Hyde R, Karran E, Christie G, Turner AJ. A human homolog of angiotensin-converting enzyme. Cloning and functional expression as a captopril-insensitive carboxypeptidase. J Biol Chem, 2000 275: p. 33238–33243. 10.1074/jbc.M002615200 10924499

[120] Tian S., et al, Characteristics of COVID-19 infection in Beijing. Journal of Infection, 2020. 10.1016/j.jinf.2020.02.018 PMC7102527 32112886

[121] Channappanavar R., et al, Sex-based differences in susceptibility to severe acute respiratory syndrome coronavirus infection. The Journal of Immunology, 2017 198(10): p. 4046–4053. 10.4049/jimmunol.1601896 28373583 PMC5450662

[122] Badawi A. and Ryoo S.G., Prevalence of comorbidities in the Middle East respiratory syndrome coronavirus (MERS-CoV): a systematic review and meta-analysis. International Journal of Infectious Diseases, 2016 49: p. 129–133. 10.1016/j.ijid.2016.06.015 27352628 PMC7110556

[123] Jaillon S., Berthenet K., and Garlanda C., Sexual dimorphism in innate immunity. Clinical reviews in allergy & immunology, 2017: p. 1–14. 10.1007/s12016-017-8648-x 28963611

[124] CDC. Symptoms of Coronavirus. 2020 12 Ooctober 2020]; Available from: https://www.cdc.gov/coronavirus/2019-ncov/symptoms-testing/symptoms.html.

[125] Sanyaolu A., et al, Comorbidity and its Impact on Patients with COVID-19. SN comprehensive clinical medicine, 2020: p. 1–8. 10.1007/s42399-020-00363-4 32838147 PMC7314621

[126] Baig A.M., et al, Evidence of the COVID-19 virus targeting the CNS: tissue distribution, host–virus interaction, and proposed neurotropic mechanisms. ACS chemical neuroscience, 2020 11(7): p. 995–998. 10.1021/acschemneuro.0c00122 32167747

[127] Netland J., et al, Severe acute respiratory syndrome coronavirus infection causes neuronal death in the absence of encephalitis in mice transgenic for human ACE2. Journal of virology, 2008 82(15): p. 7264–7275. 10.1128/JVI.00737-08 18495771 PMC2493326

[128] Ferini-Strambi L. and Salsone M., COVID-19 and neurological disorders: are neurodegenerative or neuroimmunological diseases more vulnerable? Journal of neurology, 2020: p. 1–11. 10.1007/s00415-020-10070-8 32696341 PMC7372546

[129] Rodriguez-Morales A.J., et al, Clinical, laboratory and imaging features of COVID-19: A systematic review and meta-analysis. Travel medicine and infectious disease, 2020: p. 101623 10.1016/j.tmaid.2020.101623 32179124 PMC7102608

[130] Emami A., et al, Prevalence of underlying diseases in hospitalized patients with COVID-19: a systematic review and meta-analysis. Archives of Academic Emergency Medicine, 2020 8(1). 32232218 PMC7096724

[131] Liu M., et al, Burden of diabetes, hyperglycaemia in China from to 2016: findings from the 1990 to 2016, global burden of disease study. Diabetes & metabolism, 2019 45(3): p. 286–293. 10.1016/j.diabet.2018.08.008 30196138

[132] Hu S., et al, Summary of the 2018 report on cardiovascular diseases in China. Chin Circulation J, 2019 34: p. 209.

[133] Elfiky A.A., Anti-HCV, nucleotide inhibitors, repurposing against COVID-19. Life sciences, 2020: p. 117477 10.1016/j.lfs.2020.117477 32119961 PMC7089605

[134] Wang M., et al, Remdesivir and chloroquine effectively inhibit the recently emerged novel coronavirus (2019-nCoV) in vitro. Cell research, 2020 30(3): p. 269–271. 10.1038/s41422-020-0282-0 32020029 PMC7054408

[135] Abd El-Aziz T.M. and Stockand J.D., Recent progress and challenges in drug development against COVID-19 coronavirus (SARS-CoV-2)-an update on the status. Infection, Genetics and Evolution, 2020: p. 104327 10.1016/j.meegid.2020.104327 32320825 PMC7166307

[136] Holshue M.L., et al, First case of 2019 novel coronavirus in the United States. New England Journal of Medicine, 2020. 10.1056/NEJMoa2001191 PMC7092802 32004427

[137] Gao J., Tian Z., and Yang X., Breakthrough: Chloroquine phosphate has shown apparent efficacy in treatment of COVID-19 associated pneumonia in clinical studies. Bioscience trends, 2020 10.5582/bst.2020.01047 32074550

[138] Colson P., et al, Chloroquine and hydroxychloroquine as available weapons to fight COVID-19. Int J Antimicrob Agents, 2020 105932(10.1016). 10.1016/j.ijantimicag.2020.105932 32145363 PMC7135139

[139] Jallouli M., et al, Determinants of hydroxychloroquine blood concentration variations in systemic lupus erythematosus. Arthritis & rheumatology, 2015 67(8): p. 2176–2184. 10.1002/art.39194 25989906

[140] Lee N., et al, Effects of early corticosteroid treatment on plasma SARS-associated Coronavirus RNA concentrations in adult patients. Journal of clinical virology, 2004 31(4): p. 304–309. 10.1016/j.jcv.2004.07.006 15494274 PMC7108318

[141] Lee D.T., et al, Factors associated with psychosis among patients with severe acute respiratory syndrome: a case-control study. Clinical infectious diseases, 2004 39(8): p. 1247–1249. 10.1086/424016 15486852 PMC7107870

[142] Russell C.D., Millar J.E., and Baillie J.K., Clinical evidence does not support corticosteroid treatment for 2019-nCoV lung injury. The Lancet, 2020 395(10223): p. 473–475. 10.1016/S0140-6736(20)30317-2 PMC7134694 32043983

[143] Zhai P., et al, The epidemiology, diagnosis and treatment of COVID-19. International journal of antimicrobial agents, 2020: p. 105955 10.1016/j.ijantimicag.2020.105955 32234468 PMC7138178

[144] GREIN J., OHMAGARI N., and SHIN D., original: Compassionate Use of Remdesivir for Patients with Severe Covid-19. 10.1056/NEJMoa2007016 PMC7169476 32275812

[145] NCT04252664, ClinicalTrials.gov, (2020), Feb 5.

[146] NCT04257656, ClinicalTrials.gov, (2020), Feb 6.

[147] NCT04280705, ClinicalTrials.gov, (2020), Feb 21.

[148] NCT04292730, ClinicalTrials.gov, (2020), Mar 3.

[149] NCT04292899, ClinicalTrials.gov, (2020), Mar 3.

[150] NCT04314817, ClinicalTrials.gov, (2020), Mar 19.

[151] NCT04315948, ClinicalTrials.gov, (2020), Mar 20.

[152] McKee D.L., et al, Candidate drugs against SARS-CoV-2 and COVID-19. Pharmacological Research, 2020: p. 104859 10.1016/j.phrs.2020.104859 32360480 PMC7189851

[153] Bhadra S., et al, Real-time sequence-validated loop-mediated isothermal amplification assays for detection of Middle East respiratory syndrome coronavirus (MERS-CoV). PLoS One, 2015 10(4): p. e0123126 10.1371/journal.pone.0123126 25856093 PMC4391951

[154] Huang P., et al, A rapid and specific assay for the detection of MERS-CoV. Frontiers in microbiology, 2018 9: p. 1101 10.3389/fmicb.2018.01101 29896174 PMC5987675

[155] Lee S.H., et al, One-pot reverse transcriptional loop-mediated isothermal amplification (RT-LAMP) for detecting MERS-CoV. Frontiers in microbiology, 2017 7: p. 2166 10.3389/fmicb.2016.02166 28119682 PMC5220095

[156] Du L., et al, The spike protein of SARS-CoV—a target for vaccine and therapeutic development. Nature Reviews Microbiology, 2009 7(3): p. 226–236. 10.1038/nrmicro2090 19198616 PMC2750777

[157] Diabetes T.L., COVID-19 and Racism—a double edged dagger. The Lancet. Diabetes & Endocrinology, 2020 10.1016/S2213-8587(20)30243-6 32659215 PMC7351384

[158] Gravlee C.C., Systemic racism, chronic health inequities, and COVID‐19: A syndemic in the making? American Journal of Human Biology, 2020. 10.1002/ajhb.23482 PMC7441277 32754945

[159] Bailey Z., KN (2017). Structural racism and health inequities in the USA: evidence and interventions. Lancet. 389: p. 1453–1463. 10.1016/S0140-6736(17)30569-X 28402827

[160] Laurencin C.T. and McClinton A., The COVID-19 pandemic: A call to action to identify and. 2020. 10.1007/s40615-020-00756-0 PMC7166096 32306369

[161] Johnson-Agbakwu C.E., et al, Racism, COVID-19, and Health Inequity in the USA: a Call to Action. Journal of racial and ethnic health disparities, 2020: p. 1–7. 10.1007/s40615-020-00928-y PMC7668281 33197038

[162] Ferdinand K.C. and Nasser S., Brief Review Racial/ethnic disparities in prevalence and care of patients with type 2 diabetes mellitus. 2014. 10.1185/03007995.2015.1029894 25772230

[163] Wingo S.N., Black uteri matter. 2019, LWW. 10.1097/AOG.0000000000003035 30531579

[164] Callaghan W.M. Overview of maternal mortality in the United States. in Seminars in perinatology. 2012 Elsevier 10.1053/j.semperi.2011.09.002 22280858

[165] Maddali M.M., et al, Outcomes after rigid bronchoscopy in children with suspected or confirmed foreign body aspiration: a retrospective study. Journal of Cardiothoracic and Vascular Anesthesia, 2011 25(6): p. 1005–1008. 10.1053/j.jvca.2011.02.005 21474337

[166] Song X., et al, Overall survival in patients with metastatic melanoma. Current Medical Research and Opinion, 2015 31(5): p. 987–991. 10.1185/03007995.2015.1021904 25708472

[167] Alderwick H. and Gottlieb L.M., Meanings and misunderstandings: a social determinants of health lexicon for health care systems. The Milbank Quarterly, 2019 97(2): p. 407 10.1111/1468-0009.12390 31069864 PMC6554506

[168] Price-Haywood E.G., et al, Hospitalization and mortality among black patients and white patients with Covid-19. New England Journal of Medicine, 2020 10.1056/NEJMsa2011686 32459916 PMC7269015

[169] Egede L.E. and Walker R.J., Structural racism, social risk factors, and Covid-19—a dangerous convergence for black Americans. New England Journal of Medicine, 2020 383(12): p. e77 10.1056/NEJMp2023616 32706952 PMC7747672

[170] Guan W.-j., Chen R.-c., and Zhong N.-s., Strategies for the prevention and management of coronavirus disease 2019. 2020, Eur Respiratory Soc. 10.1183/13993003.00597-2020 PMC7098484 32217658

[171] Xie C., et al, Comparison of different samples for 2019 novel coronavirus detection by nucleic acid amplification tests. International Journal of Infectious Diseases, 2020 10.1016/j.ijid.2020.02.050 32114193 PMC7129110

[172] To K.K.-W., et al, Consistent detection of 2019 novel coronavirus in saliva. Clinical Infectious Diseases, 2020 10.1093/cid/ciaa149 32047895 PMC7108139

[173] Hui J., Qing-xin K., and Hui-min W., COVID-19 prevention and control strategy: Management of close contacts in Hangzhou City, China. Journal of Infection and Public Health, 2020 10.1016/j.jiph.2020.05.007 32411309 PMC7221380

