# Supplementary material for: The prognostic value of comorbidity for the severity of COVID-19: A systematic review and meta-analysis study
Source: PLoS One. 2021 Feb 16;16(2):e0246190. doi: 10.1371/journal.pone.0246190 (PMC7886178; doi:10.1371/journal.pone.0246190)
Supplement: S1 File — (DOCX) [file pone.0246190.s003.docx]

**Boolean search strategy used to identify studies with all search terms and limits for MEDLINE:**

(((((chronic kidney disease OR renal failure [Title/Abstract]) OR (Blood pressure OR Hypertension[Title/Abstract])) OR (Diabetes[Title/Abstract])) OR (((Heart)[Title/Abstract] OR Cardio- OR cardiovascular deaths OR coronary artery disease OR cardiovascular disease OR cerebrovascular accident OR cerebrovascular disease OR heart failure )[Title/Abstract])) OR (Comorbidities[Title/Abstract])) AND (COVID[Title/Abstract])

**Table 1.** NEWCASTLE-OTTAWA quality assessment scale for cross-sectional studies

|  | selection | | | | comparability | outcome | | Total score | Total quality |
| --- | --- | --- | --- | --- | --- | --- | --- | --- | --- |
| References | 1 | 2 | 3 | 4 | 1 | 1 | 2 |  |  |
| (1) | * | * | * | ** | ** | ** | * | 10 | Very Good |
| (2) | * |  | * | ** | ** | ** |  | 8 | Good |
| (3) | * | * | * | ** | ** | ** |  | 9 | Very Good |
| (4) | * | * |  | ** | ** | ** | * | 9 | Very Good |
| (5) | * | * |  | ** | ** | ** |  | 8 | Good |
| (6) | * | * |  | ** | ** | ** | * | 9 | Very Good |
| (7) | * | * | * | ** | ** | * | * | 9 | Very Good |
| (8) | * |  | * |  |  | ** | * | 5 | satisfactory |
| (9) | * | * |  |  | ** | ** |  | 6 | satisfactory |
| (10) | * | * | * | ** | ** | ** |  | 9 | Very Good |
| (11) | * | * |  | ** | ** | ** | * | 9 | Very Good |
| (12) | * |  |  | ** | ** | ** | * | 8 | Good |
| (13) | * |  | * | ** | * | ** |  | 7 | Good |
| (14) | * | * |  | ** | ** | ** | * | 9 | Very Good |
| (15) | * |  |  | ** | * | ** | * | 7 | Good |
| (16) | * | * |  | ** | ** | ** |  | 8 | Good |
| (17) | * |  |  | ** | ** | ** |  | 6 | satisfactory |
| (18) | * |  |  | ** | ** | ** |  | 7 | Good |
| (19) | * | * |  | ** | ** | ** |  | 8 | Good |
| (20) | * |  | * | ** | ** | ** | * | 9 | Very Good |
| (21) | * |  |  | ** | * | ** |  | 6 | satisfactory |
| (22) | * | * | * | ** | * | ** | * | 9 | Very Good |
| (23) | * | * | * | ** |  | ** |  | 7 | Good |
| (24) | * |  | * | ** | ** |  |  | 6 | satisfactory |
| (25) | * |  |  | ** |  |  |  | 3 | Unsatisfactory |
| (26) | * | * |  | ** | ** | ** |  | 8 | Good |
| (27) | * |  |  | ** | * | ** | * | 7 | Good |
| (28) | * | * | * | ** | * | ** | * | 9 | Very Good |
| (29) | * | * | * | ** | ** | ** | * | 10 | Very Good |
| (30) | * | * | * | ** | ** | ** |  | 9 | Very Good |
| (31) | * |  | * | ** | ** | ** |  | 8 | Good |
| (32) | * |  | * | ** | ** | ** |  | 8 | Good |
| (33) | * | * | * | ** | ** | ** | * | 10 | Very Good |
| (34) | * | * | * | ** | * | ** | * | 9 | Very Good |
| (35) | * |  | * | ** | * | ** |  | 7 | Good |
| (36) | * | * |  | ** | ** | ** | * | 9 | Very Good |
| (37) | * |  |  | ** | * | ** |  | 6 | satisfactory |
| (38) | * | * |  | ** | ** | ** | * | 9 | Very Good |
| (39) | * |  |  | ** | ** | ** | * | 8 | Good |
| (40) | * | * |  | ** | ** | ** | * | 9 | Very Good |
| (41) | * |  | * | ** | ** | ** |  | 8 | Good |
| (42) | * | * | * | ** | * | ** | * | 9 | Very Good |
| (43) | * | * |  | ** | ** | ** | * | 9 | Very Good |
| (44) | * | * | * | ** | ** | ** |  | 9 | Very Good |
| (45) | * | * | * | ** | ** | ** |  | 9 | Very Good |
| (46) | * |  | * | ** |  | ** |  | 6 | satisfactory |
| (47) | * | * | * | ** | ** | ** | * | 10 | Very Good |
| (48) | * |  |  | ** |  | ** | * | 6 | satisfactory |
| (49) | * | * | * | ** | * | ** | * | 9 | Very Good |
| (50) | * | * | * | ** | * | ** | * | 9 | Very Good |
| (51) | * |  |  | ** |  | ** | * | 6 | satisfactory |
| (52) | * | * |  | ** | ** | ** |  | 8 | Good |
| (53) | * | * |  | ** | ** | ** | * | 9 | Very Good |
| (54) | * | * | * | ** | ** | ** | * | 10 | Very Good |
| (55) | * |  |  | ** | ** | ** | * | 8 | Good |
| (56) | * | * |  | ** | ** | ** | * | 9 | Very Good |
| (57) | * | * |  | ** | ** | ** | * | 9 | Very Good |
| (58) | * | * |  | ** | ** | ** | * | 9 | Very Good |
| (59) | * |  |  | ** | ** | ** | * | 8 | Good |
| (60) | * | * |  | ** | ** | ** | * | 9 | Very Good |
| (61) | * |  |  | ** | * | ** |  | 6 | Satisfactory |
| (62) | * | * |  | ** | ** | ** | * | 9 | Very Good |
| (63) | * | * |  | ** | ** | ** | * | 9 | Very Good |
| (64) | * |  |  | ** | * | ** |  | 6 | Satisfactory |
| (65) | * | * |  | ** | ** | ** | * | 9 | Very Good |
| (66) | * | * |  | ** | ** | ** | * | 9 | Very Good |
| (67) | * | * |  | ** | ** | ** |  | 8 | Good |
| (68) |  |  | * | ** | ** | ** | * | 8 | Good |
| (69) | * |  |  | ** |  | ** |  | 5 | Unsatisfactory |
| (70) |  |  | * | ** | ** | ** |  | 7 | Good |
| (71) |  |  | * | ** | ** | ** |  | 7 | Good |
| (72) | * | * | * | ** | ** | ** | * | 10 | Very Good |
| (73) |  |  |  | ** | ** | ** | * | 7 | Good |
| (74) |  | * | * | ** | ** | ** |  | 8 | Good |
| (75) | * |  |  | ** | * | ** |  | 6 | Satisfactory |
| (76) | * | * |  | ** | * | ** |  | 7 | Good |
| (77) | * |  |  | ** | ** | ** | * | 8 | Good |
| (78) | * | * | * | ** | ** | ** |  | 9 | Very Good |
| (79) | * | * |  | ** | ** | ** | * | 9 | Very Good |
| (80) | * | * |  | ** | ** | ** | * | 9 | Very Good |
| (81) | * | * |  | ** | ** | ** | * | 9 | Very Good |
| (82) | * | * |  | ** | ** | ** | * | 9 | Very Good |
| (83) | * | * | * | ** | ** | ** |  | 9 | Very Good |
| (84) | * | * | * | ** | ** | ** | * | 10 | Very Good |
| (85) | * | * | * | ** | ** | ** | * | 10 | Very Good |
| (86) | * | * | * | ** | ** | ** | * | 10 | Very Good |
| (87) | * | * | * | ** | ** | ** |  | 9 | Very Good |
| (88) | * | * |  | ** |  | ** |  | 6 | Satisfactory |
| (89) | * | * | * | ** | * | ** | * | 9 | Very Good |
| (90) | * | * | * | ** | ** | ** | * | 10 | Very Good |
| (91) |  | * | * | ** | * | ** | * | 8 | Good |
| (92) |  | * | * | ** | ** | ** |  | 8 | Good |
| (93) | * | * | * | ** | ** | ** | * | 10 | Very Good |
| (94) | * | * |  | ** | ** | ** |  | 8 | Good |
| (95) | * |  | * | ** | ** | ** | * | 9 | Very Good |
| (96) | * | * | * | ** | ** | ** |  | 9 | Very Good |
| (97) | * |  | * | ** | ** | ** | * | 9 | Very Good |
| (98) | * | * | * | ** | ** | ** | * | 10 | Very Good |
| (99) | * | * |  | ** | ** | ** | * | 9 | Very Good |
| (100) | * |  |  | ** | ** | ** | * | 8 | Good |
| (101) | * | * |  | ** | ** | ** | * | 9 | Very Good |
| (102) | * | * |  | ** | ** | ** |  | 8 | Good |

1. Zhou F, Yu T, Du R, Fan G, Liu Y, Liu Z, et al. Clinical course and risk factors for mortality of adult inpatients with COVID-19 in Wuhan, China: a retrospective cohort study. The lancet. 2020.

2. Du Y, Tu L, Zhu P, Mu M, Wang R, Yang P, et al. Clinical features of 85 fatal cases of COVID-19 from Wuhan: a retrospective observational study. American journal of respiratory and critical care medicine. 2020(ja).

3. Zhang J-j, Dong X, Cao Y-y, Yuan Y-d, Yang Y-b, Yan Y-q, et al. Clinical characteristics of 140 patients infected with SARS‐CoV‐2 in Wuhan, China. Allergy. 2020.

4. Guo W, Li M, Dong Y, Zhou H, Zhang Z, Tian C, et al. Diabetes is a risk factor for the progression and prognosis of COVID‐19. Diabetes/metabolism research and reviews. 2020.

5. Guan W-j, Liang W-h, Zhao Y, Liang H-r, Chen Z-s, Li Y-m, et al. Comorbidity and its impact on 1590 patients with Covid-19 in China: A Nationwide Analysis. European Respiratory Journal. 2020.

6. Wu C, Chen X, Cai Y, Zhou X, Xu S, Huang H, et al. Risk factors associated with acute respiratory distress syndrome and death in patients with coronavirus disease 2019 pneumonia in Wuhan, China. JAMA internal medicine. 2020.

7. Wang L, He W, Yu X, Hu D, Bao M, Liu H, et al. Coronavirus Disease 2019 in elderly patients: characteristics and prognostic factors based on 4-week follow-up. Journal of Infection. 2020.

8. Li X, Wang L, Yan S, Yang F, Xiang L, Zhu J, et al. Clinical characteristics of 25 death cases with COVID-19: a retrospective review of medical records in a single medical center, Wuhan, China. International Journal of Infectious Diseases. 2020.

9. Chow N, Fleming-Dutra K, Gierke R, Hall A, Hughes M, Pilishvili T, et al. Preliminary estimates of the prevalence of selected underlying health conditions among patients with coronavirus disease 2019—United States, February 12–March 28, 2020. 2020.

10. Garg S. Hospitalization rates and characteristics of patients hospitalized with laboratory-confirmed coronavirus disease 2019—COVID-NET, 14 States, March 1–30, 2020. MMWR Morbidity and Mortality Weekly Report. 2020;69.

11. Wang X, Fang J, Zhu Y, Chen L, Ding F, Zhou R, et al. Clinical characteristics of non-critically ill patients with novel coronavirus infection (COVID-19) in a Fangcang Hospital. Clinical Microbiology and Infection. 2020.

12. Huang C, Wang Y, Li X, Ren L, Zhao J, Hu Y, et al. Clinical features of patients infected with 2019 novel coronavirus in Wuhan, China. The lancet. 2020;395(10223):497-506.

13. Chen N, Zhou M, Dong X, Qu J, Gong F, Han Y, et al. Epidemiological and clinical characteristics of 99 cases of 2019 novel coronavirus pneumonia in Wuhan, China: a descriptive study. The Lancet. 2020;395(10223):507-13.

14. Wang D, Hu B, Hu C, Zhu F, Liu X, Zhang J, et al. Clinical characteristics of 138 hospitalized patients with 2019 novel coronavirus–infected pneumonia in Wuhan, China. Jama. 2020;323(11):1061-9.

15. Li J, Li S, Cai Y, Liu Q, Li X, Zeng Z, et al. Epidemiological and Clinical Characteristics of 17 Hospitalized Patients with 2019 Novel Coronavirus Infections Outside Wuhan, China. medRxiv. 2020.

16. Guan W-j, Ni Z-y, Hu Y, Liang W-h, Ou C-q, He J-x, et al. Clinical characteristics of coronavirus disease 2019 in China. New England journal of medicine. 2020;382(18):1708-20.

17. Xu X-W, Wu X-X, Jiang X-G, Xu K-J, Ying L-J, Ma C-L, et al. Clinical findings in a group of patients infected with the 2019 novel coronavirus (SARS-Cov-2) outside of Wuhan, China: retrospective case series. bmj. 2020;368.

18. Wu J, Liu J, Zhao X, Liu C, Wang W, Wang D, et al. Clinical Characteristics of Imported Cases of Coronavirus Disease 2019 (COVID-19) in Jiangsu Province: A Multicenter Descriptive Study. Clinical Infectious Diseases. 2020.

19. Liu K, Fang Y-Y, Deng Y, Liu W, Wang M-F, Ma J-P, et al. Clinical characteristics of novel coronavirus cases in tertiary hospitals in Hubei Province. Chinese medical journal. 2020.

20. Liu J, Liu Y, Xiang P, Pu L, Xiong H, Li C, et al. Neutrophil-to-lymphocyte ratio predicts severe illness patients with 2019 novel coronavirus in the early stage. MedRxiv. 2020.

21. Yang X, Yu Y, Xu J, Shu H, Liu H, Wu Y, et al. Clinical course and outcomes of critically ill patients with SARS-CoV-2 pneumonia in Wuhan, China: a single-centered, retrospective, observational study. The Lancet Respiratory Medicine. 2020.

22. Guo T, Fan Y, Chen M, Wu X, Zhang L, He T, et al. Cardiovascular implications of fatal outcomes of patients with coronavirus disease 2019 (COVID-19). JAMA cardiology. 2020.

23. Onder G, Rezza G, Brusaferro S. Case-fatality rate and characteristics of patients dying in relation to COVID-19 in Italy. Jama. 2020.

24. Liu Y, Yang Y, Zhang C, Huang F, Wang F, Yuan J, et al. Clinical and biochemical indexes from 2019-nCoV infected patients linked to viral loads and lung injury. Science China Life Sciences. 2020;63(3):364-74.

25. Zhang MQ, Wang XH, Chen YL, Zhao KL, Cai YQ, An CL, et al. [Clinical features of 2019 novel coronavirus pneumonia in the early stage from a fever clinic in Beijing]. Zhonghua jie he he hu xi za zhi = Zhonghua jiehe he huxi zazhi = Chinese journal of tuberculosis and respiratory diseases. 2020;43(0):E013.

26. Surveillances V. The epidemiological characteristics of an outbreak of 2019 novel coronavirus diseases (COVID-19)—China, 2020. China CDC Weekly. 2020;2(8):113-22.

27. Liu W, Tao Z-W, Wang L, Yuan M-L, Liu K, Zhou L, et al. Analysis of factors associated with disease outcomes in hospitalized patients with 2019 novel coronavirus disease. Chinese medical journal. 2020.

28. Cheng Y, Luo R, Wang K, Zhang M, Wang Z, Dong L, et al. Kidney disease is associated with in-hospital death of patients with COVID-19. Kidney International. 2020.

29. Li Z, Wu M, Yao J, Guo J, Liao X, Song S, et al. Caution on kidney dysfunctions of COVID-19 patients. 2020.

30. Chen T, Wu D, Chen H, Yan W, Yang D, Chen G, et al. Clinical characteristics of 113 deceased patients with coronavirus disease 2019: retrospective study. Bmj. 2020;368.

31. Zhang B, Zhou X, Qiu Y, Feng F, Feng J, Jia Y, et al. Clinical characteristics of 82 death cases with COVID-19. medRxiv. 2020.

32. Zhang H, Chen Y, Yuan Q, Xia Q-X, Zeng X-P, Peng J-T, et al. Identification of kidney transplant recipients with coronavirus disease 2019. European Urology. 2020.

33. Shi S, Qin M, Shen B, Cai Y, Liu T, Yang F, et al. Association of cardiac injury with mortality in hospitalized patients with COVID-19 in Wuhan, China. JAMA cardiology. 2020.

34. Wang L, Li X, Chen H, Yan S, Li D, Li Y, et al. Coronavirus disease 19 infection does not result in acute kidney injury: an analysis of 116 hospitalized patients from Wuhan, China. American journal of nephrology. 2020;51(5):343-8.

35. Arentz M, Yim E, Klaff L, Lokhandwala S, Riedo FX, Chong M, et al. Characteristics and outcomes of 21 critically ill patients with COVID-19 in Washington State. Jama. 2020.

36. Chen T, Dai Z, Mo P, Li X, Ma Z, Song S, et al. Clinical characteristics and outcomes of older patients with coronavirus disease 2019 (COVID-19) in Wuhan, China (2019): a single-centered, retrospective study. The Journals of Gerontology: Series A. 2020.

37. Qian G-Q, Yang N-B, Ding F, Ma AHY, Wang Z-Y, Shen Y-F, et al. Epidemiologic and Clinical Characteristics of 91 Hospitalized Patients with COVID-19 in Zhejiang, China: A retrospective, multi-centre case series. QJM: An International Journal of Medicine. 2020.

38. Qin C, Zhou L, Hu Z, Zhang S, Yang S, Tao Y, et al. Dysregulation of immune response in patients with COVID-19 in Wuhan, China. Clinical Infectious Diseases. 2020.

39. Wang Z, Yang B, Li Q, Wen L, Zhang R. Clinical Features of 69 Cases with Coronavirus Disease 2019 in Wuhan, China. Clinical infectious diseases : an official publication of the Infectious Diseases Society of America. 2020.

40. Zhang G, Hu C, Luo L, Fang F, Chen Y, Li J, et al. Clinical features and short-term outcomes of 221 patients with COVID-19 in Wuhan, China. Journal of Clinical Virology. 2020:104364.

41. Xu X, Yu C, Qu J, Zhang L, Jiang S, Huang D, et al. Imaging and clinical features of patients with 2019 novel coronavirus SARS-CoV-2. European journal of nuclear medicine and molecular imaging. 2020:1-6.

42. Du R-H, Liu L-M, Yin W, Wang W, Guan L-L, Yuan M-L, et al. Hospitalization and Critical Care of 109 Decedents with COVID-19 Pneumonia in Wuhan, China. Annals of the American Thoracic Society. 2020(ja).

43. Cao J, Tu W-J, Cheng W, Yu L, Liu Y-K, Hu X, et al. Clinical Features and Short-term Outcomes of 102 Patients with Corona Virus Disease 2019 in Wuhan, China. Clinical Infectious Diseases. 2020.

44. Grasselli G, Zangrillo A, Zanella A, Antonelli M, Cabrini L, Castelli A, et al. Baseline characteristics and outcomes of 1591 patients infected with SARS-CoV-2 admitted to ICUs of the Lombardy Region, Italy. Jama. 2020.

45. Richardson S, Hirsch JS, Narasimhan M, Crawford JM, McGinn T, Davidson KW, et al. Presenting Characteristics, Comorbidities, and Outcomes Among 5700 Patients Hospitalized With COVID-19 in the New York City Area. JAMA. 2020.

46. He XW, Lai JS, Cheng J, Wang MW, Liu YJ, Xiao ZC, et al. [Impact of complicated myocardial injury on the clinical outcome of severe or critically ill COVID-19 patients]. Zhonghua xin xue guan bing za zhi. 2020;48(0):E011.

47. Fu L, Fei J, Xiang H-X, Xiang Y, Tan Z-X, Li M-D, et al. Influence factors of death risk among COVID-19 patients in Wuhan, China: a hospital-based case-cohort study. medRxiv. 2020:2020.03.13.20035329.

48. Han Y, Zhang H, Mu S, Wei W, Jin C, Xue Y, et al. Lactate dehydrogenase, a Risk Factor of Severe COVID-19 Patients. medRxiv. 2020.

49. Zhang F, Yang D, Li J, Gao P, Chen T, Cheng Z, et al. Myocardial injury is associated with in-hospital mortality of confirmed or suspected COVID-19 in Wuhan, China: A single center retrospective cohort study. MedRxiv. 2020.

50. Hu L, Chen S, Fu Y, Gao Z, Long H, Ren H-w, et al. Risk factors associated with clinical outcomes in 323 COVID-19 patients in Wuhan, China. Medrxiv. 2020.

51. Ji D, Zhang D, Chen Z, Xu Z, Zhao P, Zhang M, et al. Clinical Characteristics Predicting Progression of COVID-19. 2020.

52. Cai Q, Huang D, Ou P, Yu H, Zhu Z, Xia Z, et al. 2019-nCoV Pneumonia in a Normal Work Infectious Diseases Hospital Besides Hubei Province, China. 2020.

53. Lu H, Ai J, Shen Y, Li Y, Li T, Zhou X, et al. A descriptive study of the impact of diseases control and prevention on the epidemics dynamics and clinical features of SARS-CoV-2 outbreak in Shanghai, lessons learned for metropolis epidemics prevention. medRxiv. 2020.

54. Wang G, Wu C, Zhang Q, Wu F, Yu B, Lv J, et al. Epidemiological and Clinical Features of Corona Virus Disease 2019 (COVID-19) in Changsha, China. China (3/1/2020). 2020.

55. Ma K-L, Liu Z-H, Cao C-f, Liu M-K, Liao J, Zou J-B, et al. COVID-19 myocarditis and severity factors: an adult cohort study. medRxiv. 2020.

56. Liu Y, Sun W, Li J, Chen L, Wang Y, Zhang L, et al. Clinical features and progression of acute respiratory distress syndrome in coronavirus disease 2019. MedRxiv. 2020.

57. Li X, Xu S, Yu M, Wang K, Tao Y, Zhou Y, et al. Risk factors for severity and mortality in adult COVID-19 inpatients in Wuhan. Journal of Allergy and Clinical Immunology. 2020.

58. Xu K, Chen Y, Yuan J, Yi P, Ding C, Wu W, et al. Factors associated with prolonged viral RNA shedding in patients with COVID-19. Clinical Infectious Diseases. 2020.

59. Yuan M, Yin W, Tao Z, Tan W, Hu Y. Association of radiologic findings with mortality of patients infected with 2019 novel coronavirus in Wuhan, China. PloS one. 2020;15(3):e0230548.

60. Lian J, Jin X, Hao S, Cai H, Zhang S, Zheng L, et al. Analysis of epidemiological and clinical features in older patients with coronavirus disease 2019 (COVID-19) outside Wuhan. Clinical infectious diseases. 2020;71(15):740-7.

61. Escalera-Antezana JP, Lizon-Ferrufino NF, Maldonado-Alanoca A, Alarcón-De-la-Vega G, Alvarado-Arnez LE, Balderrama-Saavedra MA, et al. Clinical features of cases and a cluster of Coronavirus Disease 2019 (COVID-19) in Bolivia imported from Italy and Spain. Travel Medicine and Infectious Disease. 2020:101653.

62. Zheng F, Tang W, Li H, Huang Y, Xie Y, Zhou Z. Clinical characteristics of 161 cases of corona virus disease 2019 (COVID-19) in Changsha. Eur Rev Med Pharmacol Sci. 2020;24(6):3404-10.

63. Wan S, Xiang Y, Fang W, Zheng Y, Li B, Hu Y, et al. Clinical features and treatment of COVID‐19 patients in northeast Chongqing. Journal of medical virology. 2020.

64. Zhao S, Ling K, Yan H, Zhong L, Peng X, Yao S, et al. Anesthetic management of patients with suspected 2019 novel coronavirus infection during emergency procedures. Journal of cardiothoracic and vascular anesthesia. 2020.

65. Mao L, Jin H, Wang M, Hu Y, Chen S, He Q, et al. Neurologic manifestations of hospitalized patients with coronavirus disease 2019 in Wuhan, China. JAMA neurology. 2020;77(6):683-90.

66. Deng Q, Hu B, Zhang Y, Wang H, Zhou X, Hu W, et al. Suspected myocardial injury in patients with COVID-19: Evidence from front-line clinical observation in Wuhan, China. International journal of cardiology. 2020.

67. Shao F, Xu S, Ma X, Xu Z, Lyu J, Ng M, et al. In-hospital cardiac arrest outcomes among patients with COVID-19 pneumonia in Wuhan, China. Resuscitation. 2020.

68. Tu W-J, Cao J, Yu L, Hu X, Liu Q. Clinicolaboratory study of 25 fatal cases of COVID-19 in Wuhan. Intensive care medicine. 2020:1-4.

69. Young BE, Ong SWX, Kalimuddin S, Low JG, Tan SY, Loh J, et al. Epidemiologic features and clinical course of patients infected with SARS-CoV-2 in Singapore. Jama. 2020;323(15):1488-94.

70. Park S, Lee M, Kim S, Kwak Y, Kwon K, Park J, et al. Analysis on 54 mortality cases of coronavirus disease 2019 in the Republic of Korea from January 19 to March 10, 2020. J Korean Med Sci. 2020;35(12):e132.

71. Yang F, Shi S, Zhu J, Shi J, Dai K, Chen X. Analysis of 92 deceased patients with COVID‐19. Journal of medical virology. 2020.

72. Meng Y, Wu P, Lu W, Liu K, Ma K, Huang L, et al. Sex-specific clinical characteristics and prognosis of coronavirus disease-19 infection in Wuhan, China: A retrospective study of 168 severe patients. PLoS pathogens. 2020;16(4):e1008520.

73. Li Y-K, Peng S, Li L-Q, Wang Q, Ping W, Zhang N, et al. Clinical and transmission characteristics of Covid-19—a retrospective study of 25 cases from a single thoracic surgery department. Current medical science. 2020:1-6.

74. Lovell N, Maddocks M, Etkind SN, Taylor K, Carey I, Vora V, et al. Characteristics, symptom management and outcomes of 101 patients with COVID-19 referred for hospital palliative care. Journal of Pain and Symptom Management. 2020.

75. Liu C, Wu C, Zheng X, Zeng F, Liu J, Wang P, et al. Clinical features and multidisciplinary treatment outcome of COVID-19 pneumonia: A report of three cases. Journal of the Formosan Medical Association. 2020.

76. Yang W, Cao Q, Qin L, Wang X, Cheng Z, Pan A, et al. Clinical characteristics and imaging manifestations of the 2019 novel coronavirus disease (COVID-19): A multi-center study in Wenzhou city, Zhejiang, China. Journal of Infection. 2020.

77. Shi H, Han X, Jiang N, Cao Y, Alwalid O, Gu J, et al. Radiological findings from 81 patients with COVID-19 pneumonia in Wuhan, China: a descriptive study. The Lancet Infectious Diseases. 2020.

78. Choe YJ. Coronavirus disease-19: The First 7,755 Cases in the Republic of Korea. medRxiv. 2020.

79. Yao Q, Wang P, Wang X, Qie G, Meng M, Tong X, et al. Retrospective study of risk factors for severe SARS-Cov-2 infections in hospitalized adult patients. Polish archives of internal medicine. 2020.

80. Feng Y, Ling Y, Bai T, Xie Y, Huang J, Li J, et al. COVID-19 with different severities: a multicenter study of clinical features. American journal of respiratory and critical care medicine. 2020;201(11):1380-8.

81. Mo P, Xing Y, Xiao Y, Deng L, Zhao Q, Wang H, et al. Clinical characteristics of refractory COVID-19 pneumonia in Wuhan, China. Clinical Infectious Diseases. 2020.

82. Shi Y, Yu X, Zhao H, Wang H, Zhao R, Sheng J. Host susceptibility to severe COVID-19 and establishment of a host risk score: findings of 487 cases outside Wuhan. Critical Care. 2020;24(1):1-4.

83. Mancia G, Rea F, Ludergnani M, Apolone G, Corrao G. Renin–angiotensin–aldosterone system blockers and the risk of Covid-19. New England Journal of Medicine. 2020.

84. Mehta N, Kalra A, Nowacki AS, Anjewierden S, Han Z, Bhat P, et al. Association of use of angiotensin-converting enzyme inhibitors and angiotensin II receptor blockers with testing positive for coronavirus disease 2019 (COVID-19). JAMA cardiology. 2020.

85. Bean D, Kraljevic Z, Searle T, Bendayan R, Pickles A, Folarin A, et al. ACE-inhibitors and Angiotensin-2 Receptor Blockers are not associated with severe SARS-COVID19 infection in a multi-site UK acute Hospital Trust. MedRxiv. 2020.

86. Caraballo C, McCullough M, Fuery M, Chouairi F, Keating C, Ravindra N, et al. COVID-19 Infections and Outcomes in a Live Registry of Heart Failure Patients Across an Integrated Health Care System. medRxiv. 2020.

87. de Abajo FJ, Rodríguez-Martín S, Lerma V, Mejía-Abril G, Aguilar M, García-Luque A, et al. Use of renin–angiotensin–aldosterone system inhibitors and risk of COVID-19 requiring admission to hospital: a case-population study. The Lancet. 2020.

88. De Spiegeleer A, Bronselaer A, Teo JT, Byttebier G, De Tre G, Belmans L, et al. The effects of ARBs, ACEIs and statins on clinical outcomes of COVID-19 infection among nursing home residents. medRxiv. 2020.

89. Ebinger JE, Achamallah N, Ji H, Claggett BL, Sun N, Botting P, et al. Pre-Existing Characteristics Associated with Covid-19 Illness Severity. medRxiv. 2020.

90. Khawaja AP, Warwick AN, Hysi PG, Kastner A, Dick A, Khaw PT, et al. Associations with covid-19 hospitalisation amongst 406,793 adults: the UK Biobank prospective cohort study. medRxiv. 2020.

91. Raisi-Estabragh Z, McCracken C, Ardissino M, Bethell MS, Cooper J, Cooper C, et al. NON-WHITE ETHNICITY, MALE SEX, AND HIGHER BODY MASS INDEX, BUT NOT MEDICATIONS ACTING ON THE RENIN-ANGIOTENSIN SYSTEM ARE ASSOCIATED WITH CORONAVIRUS DISEASE 2019 (COVID-19) HOSPITALISATION: REVIEW OF THE FIRST 669 CASES FROM THE UK BIOBANK. medRxiv. 2020.

92. McMichael TM, Currie DW, Clark S, Pogosjans S, Kay M, Schwartz NG, et al. Epidemiology of Covid-19 in a long-term care facility in King County, Washington. New England Journal of Medicine. 2020;382(21):2005-11.

93. Fabio C, Antonella C, Patrizia R-Q, Annalisa R, Laura G, Caterina C, et al. Early predictors of clinical outcomes of COVID-19 outbreak in Milan, Italy. Clinical Immunology. 2020:108509.

94. Duanmu Y, Brown IP, Gibb WR, Singh J, Matheson LW, Blomkalns AL, et al. Characteristics of Emergency Department Patients With COVID‐19 at a Single Site in Northern California: Clinical Observations and Public Health Implications. Academic Emergency Medicine. 2020.

95. Inciardi RM, Adamo M, Lupi L, Cani DS, Di Pasquale M, Tomasoni D, et al. Characteristics and outcomes of patients hospitalized for COVID-19 and cardiac disease in Northern Italy. European heart journal. 2020;41(19):1821-9.

96. Buckner FS, McCulloch DJ, Atluri V, Blain M, McGuffin SA, Nalla AK, et al. Clinical Features and Outcomes of 105 Hospitalized patients with COVID-19 in Seattle, Washington. Clinical Infectious Diseases. 2020.

97. Halvatsiotis P, Kotanidou A, Tzannis K, Jahaj E, Magira E, Theodorakopoulou M, et al. Demographic and clinical features of critically ill patients with COVID-19 in Greece: The burden of diabetes and obesity. Diabetes research and clinical practice. 2020;166:108331.

98. Colombi D, Bodini FC, Petrini M, Maffi G, Morelli N, Milanese G, et al. Well-aerated lung on admitting chest CT to predict adverse outcome in COVID-19 pneumonia. Radiology. 2020:201433.

99. Marcello RK, Dolle J, Grami S, Adule R, Li Z, Tatem K, et al. Characteristics and Outcomes of COVID-19 Patients in New York City's Public Hospital System. medRxiv. 2020.

100. Li K, Wu J, Wu F, Guo D, Chen L, Fang Z, et al. The clinical and chest CT features associated with severe and critical COVID-19 pneumonia. Investigative radiology. 2020.

101. Wang K, Zuo P, Liu Y, Zhang M, Zhao X, Xie S, et al. Clinical and laboratory predictors of in-hospital mortality in patients with COVID-19: a cohort study in Wuhan, China. Clinical infectious diseases. 2020.

102. Liang W, Liang H, Ou L, Chen B, Chen A, Li C, et al. Development and validation of a clinical risk score to predict the occurrence of critical illness in hospitalized patients with COVID-19. JAMA Internal Medicine. 2020.
